# Pharmacokinetics and Pharmacodynamics of Intensive Antituberculosis Treatment of Tuberculous Meningitis

**DOI:** 10.1002/cpt.1783

**Published:** 2020-02-29

**Authors:** Junjie Ding, Nguyen Thuy Thuong Thuong, Toi Van Pham, Dorothee Heemskerk, Thomas Pouplin, Chau Thi Hong Tran, Mai Thi Hoang Nguyen, Phu Hoan Nguyen, Loc Phu Phan, Chau Van Vinh Nguyen, Guy Thwaites, Joel Tarning

**Affiliations:** ^1^ Nuffield Department of Clinical Medicine Centre for Tropical Medicine and Global Health University of Oxford Oxford UK; ^2^ The WorldWide Antimalarial Resistance Network Oxford UK; ^3^ Mahidol‐Oxford Tropical Medicine Research Unit Faculty of Tropical Medicine Mahidol University Bangkok Thailand; ^4^ Oxford University Clinical Research Unit Centre for Tropical Medicine Ho Chi Minh City Vietnam; ^5^ Hospital for Tropical Diseases Ho Chi Minh City Vietnam

## Abstract

The most effective antituberculosis drug treatment regimen for tuberculous meningitis is uncertain. We conducted a randomized controlled trial comparing standard treatment with a regimen intensified by rifampin 15 mg/kg and levofloxacin for the first 60 days. The intensified regimen did not improve survival or any other outcome. We therefore conducted a nested pharmacokinetic/pharmacodynamic study in 237 trial participants to define exposure–response relationships that might explain the trial results and improve future therapy. Rifampin 15 mg/kg increased plasma and cerebrospinal fluid (CSF) exposures compared with 10 mg/kg: day 14 exposure increased from 48.2 hour·mg/L (range 18.2–93.8) to 82.5 hour·mg/L (range 8.7–161.0) in plasma and from 3.5 hour·mg/L (range 1.2–9.6) to 6.0 hour·mg/L (range 0.7–15.1) in CSF. However, there was no relationship between rifampin exposure and survival. In contrast, we found that isoniazid exposure was associated with survival, with low exposure predictive of death, and was linked to a fast metabolizer phenotype. Higher doses of isoniazid should be investigated, especially in fast metabolizers.

**Study Highlights**

**WHAT IS THE CURRENT KNOWLEDGE ON THE TOPIC?**

☑ The best tuberculous meningitis (TBM) treatment is not well characterized. Higher rifampin exposure has been reported to be associated with greater survival. However, our recent trial in 817 patients with TBM failed to demonstrate benefit on survival of higher dose rifampin (15 mg/kg/day vs. 10 mg/kg/day).

**WHAT QUESTION DID THIS STUDY ADDRESS?**

☑ What are the exposure–response relationships for antituberculosis drugs in the treatment of TBM and should the dose of rifampin, or other drugs, be increased to prevent death?

**WHAT DOES THIS STUDY ADD TO OUR KNOWLEDGE?**

☑ Pharmacokinetic properties of rifampin, isoniazid, pyrazinamide, ethambutol, and levofloxacin in plasma and cerebrospinal fluid were characterized in 237 patients with TBM. A time‐to‐event model was used to describe the risk of death. After adjustment for Glasgow coma scale and HIV, rifampin exposure was not linked to survival. However, high isoniazid exposures were strongly associated with reduced hazard of death.

**HOW MIGHT THIS CHANGE CLINICAL PHARMACOLOGY OR TRANSLATIONAL SCIENCE?**

☑ Higher doses of isoniazid for the treatment of TBM should be investigated, especially in fast acetylators.

The optimal antituberculosis chemotherapy of tuberculous meningitis (TBM) is poorly defined.^1^ The current choice of drugs and their dosing is based upon the treatment of pulmonary tuberculosis, but the distribution of some of the drugs into the brain is restricted by the blood‐brain barrier, which may result in suboptimal drug exposures, bacterial killing, and treatment outcomes. Thus, it is hypothesized that bacterial killing and clinical outcomes from TBM can be improved by increasing the doses of some drugs, in particular rifampin, and by using drugs which cross the blood‐brain barrier freely.^2^

We recently tested this hypothesis in a randomized controlled trial comparing the standard four‐drug antituberculosis regimen (using 10 mg/kg/day rifampin) with a five‐drug "intensive" regimen that included higher dose rifampin (15 mg/kg/day) and the addition of levofloxacin (1,000 mg/kg/day) for the first 2 months of TBM treatment.^3^ The trial randomized 817 Vietnamese adults with TBM 1:1 to the two regimens, but did not demonstrate any benefit of the intensive regimen on survival or any other endpoint.

The trial’s negative results need an explanation. Recent studies in pulmonary tuberculosis treatment suggest that much higher doses of rifampin (up to 40 mg/kg/day) can be given safely and result in increased drug exposure and bacterial killing.^4^ Clinical and pharmacological studies conducted in Indonesian adults with TBM have suggested that survival increases with increased drug exposure and rifampin doses of at least 20 mg/kg, and possibly up to 35 mg/kg/day, may be required to improve outcomes.^5^, ^6^, ^7^, ^8^ Taken together, these studies suggest the rifampin dose used in our trial (15 mg/kg/day) may have been too low.

There are, however, other possible explanations. Possibly, the contribution to (early) bacterial killing of other drugs may have moderated the effect of higher dose rifampicin and levofloxacin. Alternatively, outcome from TBM is intimately associated with intracerebral inflammation,^9^ and enhanced bacterial killing might provoke increased intracerebral inflammation, which may offset any beneficial effect of more rapid bacterial clearance and may even paradoxically worsen rather than improve outcomes.

The objective of the current study was to define the plasma and cerebrospinal fluid (CSF) pharmacokinetics (PK) of rifampin, isoniazid, pyrazinamide, ethambutol, and levofloxacin in a subset of 237 patients enrolled into the trial and to investigate the relationships between drug exposures and survival (pharmacokinetics/pharmacodynamics; PK/PD). Our aim was to better understand the trial results and explore whether there was evidence for dose–response relationships that would support the need for higher doses of rifampin or the addition of other drugs with better brain penetration.

## Methods

### Study design and drug regimen

The study design and inclusion criteria have been previously described.^3^ In brief, a randomized, double‐blind, placebo‐controlled trial involving HIV–infected and HIV‐uninfected adults with a clinical diagnosis of TBM was conducted at two Vietnamese hospitals. The trial aim was to compare the efficacy of standard dose, 9‐month antituberculosis regimen with an intensified regimen that included higher dose rifampin (15 mg/kg/day) and levofloxacin for the first 8 weeks of treatment. The primary outcome was death by 9 months after randomization. The International Standard Randomized Controlled Trial (ISRCT) registered number was ISRCTN61649292.

All patients received standard oral antituberculosis treatment: Isoniazid (5 mg/kg/day; maximum, 300 mg/day), rifampin (10 mg/kg/day), pyrazinamide (25 mg/kg/day; maximum, 2 g/day), and ethambutol (20 mg/kg/day; maximum, 1.2 g/day) for 3 months, followed by rifampin and isoniazid at the same doses for an additional 6 months. Intensified treatment consisted of the standard 9‐month regimen with the addition of levofloxacin (20 mg/kg, per day) and an increased dose of rifampin (15 mg/kg/day) for the first 8 weeks.

Patients who had previously received antituberculosis treatment received streptomycin (20 mg/kg per day; maximum, 1 g/day) for the first 3 months. HIV‐infected patients received efavirenz‐based antiretroviral treatment in accordance with Vietnamese guidelines. All patients received adjunctive treatment with dexamethasone for the first 6–8 weeks.

A total of 817 patients (349 HIV infected) were enrolled; 409 and 408 patients were randomly assigned to standard regimen arm and intensified treatment arm, respectively. A PK/pharmacodynamic (PD) substudy was designed to investigate the relationship between antituberculosis drug exposure and treatment responses. Two hundred thirty‐seven patients consecutively enrolled into the trial at one center (Hospital for Tropical Diseases) were entered into the PK/PD substudy.

### Blood samples

Sixty patients, stratified by treatment arm and HIV status (15 in each subgroup), had intensive sampling, where blood samples were taken at steady state (14 days after initiation of treatment) 0 (predose), 0.5, 1, 2, 3, 4, 6, 8, and 12 hours after the dose.

CSF (> 5 mL) was sampled after 1, 2, and 9 months of antituberculosis treatment. The timing of the sample was randomly designated at 0–3 hours, 3–6 hours, or 6–12 hours after the antituberculosis drugs were taken. An additional CSF sample at month 3 was taken for HIV‐infected patients. A paired blood sample (3 mL) was taken with each CSF sample. Further details concerning blood sampling, storage, and transportation are provided in the **Supplementary Material**.

### Concentration quantification

Plasma and CSF concentrations of rifampin, isoniazid, and the plasma concentration of pyrazinamide and ethambutol were measured, using a liquid chromatography with tandem mass spectrometry–based assay, and the plasma and CSF concentrations of levofloxacin were measured using high performance liquid chromatography with fluorescence detection, validated according to US Food and Drug Administration (FDA) guidelines. Full details can be found in the **Supplementary Material**.

### Data collection

The information about each participant’s age, bodyweight, height, gender, HIV status, dose regimen (amount, date, time), Glasgow coma scale (GCS), MRC disease severity grade (1–3), hematology assays (white cell count, hemoglobin, platelets), biochemistry assays (albumin, creatinine, bilirubin, aspartate transaminase (AST), alanine aminotransferase (ALT), and blood glucose), CSF assays (white blood cell, glucose, protein, and lactate), as well as the clinical outcome (death and time) were collected. Moreover, the CSF culture results and their minimum inhibitory concentration (MIC) values for isoniazid and rifampin were recorded. The creatinine clearance for each patient was calculated using Cockcroft‐Gault equation, based on gender, age, bodyweight, and serum creatinine level. The sampling date and time were collected in the field and added to the plasma and/or CSF concentrations of antituberculosis drugs after the assays were performed.

### PK–PD analysis

Population PK/PD analyses were performed using nonlinear mixed‐effects modeling in the software NONMEM (version 7.4, ICON Development Solutions, Ellicott City, MD). Full details can be found in the **Supplementary Material**.

### PK modeling

An enzyme turnover model was used to account for the auto‐induction process of rifampin, as previously suggested.^4^, ^10^, ^11^ Due to lack of blood samples at the initial stage of auto‐induction (e.g., day 1 after the treatment), the parameters related to the auto‐induction process (the maximum increase in enzyme formation rate (E_max_), enzyme degradation rate, and concentration corresponding to 50% of E_max_ (EC_50_)), were fixed to previously reported values.^4^

Moreover, rifampin has been reported to show a dose‐dependent bioavailability,^4^ and this was unconditionally added on relative bioavailability as described in Eq. 1.

(1) $$F=\left(1+\frac{F_{\max}\times \left(\text{Dose}-450\right)}{{\text{ED}}_{50}+\left(\text{Dose}-450\right)}\right)$$

where *F*_max_ (0.504) is the maximal increase in relative bioavailability and ED_50_ (67 mg) refers to the dose corresponding to half the F_max_.

Isoniazid is mainly metabolized by the N‐acetyltransferase 2 (NAT2) enzyme^12^. NAT2 is polymorphic and classified as slow, intermediate, or fast acetylators phenotype. A mixture model applied on clearance was used to characterize the effect of metabolic polymorphism on isoniazid elimination and defining subjects as either fast or slow eliminators.^13^

Fat‐free mass (FFM) (derived from sex, bodyweight, and body mass index^14^ as suggested in recent studies^4^, ^15^) and total bodyweight were allometrically scaled to all clearance parameters (Eq. 2), and volume of distribution parameters (Eq. 3), and evaluated for all drugs. FFW_i_ is the individual FFM. FFM was chosen if there was no significant difference between the two parameterizations, because of recent literature suggesting FFM to be superior to total body weight as a PK body size descriptor.

(2) $$\theta_{i}=\theta \cdot {\left(\frac{{\text{FFM}}_{i}}{70}\right)}^{0.75}\cdot \text{exp(}\eta_{i,\theta })$$

(3) $$\theta_{i}=\theta \cdot \left(\frac{{\text{FFW}}_{i}}{70}\right)\cdot \exp\left(\eta_{i,\theta }\right)$$

Then, Pearson’s correlation tests and analysis of variance were used to investigate correlation between random effects of parameters and continuous and categorical covariates, respectively. Significant covariates (*P* < 0.05) were investigated formally on model parameters using a forward selection (*P* = 0.05) and backward elimination (*P* = 0.01) approach in NONMEM.

### PD modeling

PD outcomes were modeled using a time‐to‐event approach. Different parametric hazard distributions, including exponential, Gompertz, Weibull, and log‐normal functions, were evaluated for the best description of the distribution of time‐to‐death. The covariate selection process started with a univariate analysis (analysis of variance or Pearson’s correlation test) of each clinical variable, including baseline GCS, HIV status, biochemistry assays, and CSF assays. Any clinical covariate having a significant univariate test (*P* < 0.01) was selected for a multivariate analysis using NONMEM. The individual CSF and/or plasma exposure (area under the concentration‐time curve (AUC) and/or peak concentration (C_max_)) at steady state for each antituberculosis drug were obtained from the Empirical Bayes *post hoc* estimates from the final population PK model. The relationship between drug exposure and total hazard were investigated using either linear, E_max_ or sigmoidal E_max_ models.

The classification and regression tree (CART) analysis, using a recursive partitioning algorithm, was employed to verify the covariates identified by NONMEM and their optimal cutoff points. CART analyses were performed using the rpart package in R language (version 3.4.1, the R Foundation for Statistical Computing, Vienna, Austria). The general process in rpart analysis was first to grow a complex tree and then prune the tree back by cross‐validation. CART proceeds recursively until the stopping rules are reached, that the number of observations in any terminal node is less than prespecific value (20 in this study) or the node is pure. The following model specifications were considered in the CART^16^: (i) cost‐complexity parameter was set to be 0.01; and (ii) validation was performed by 10‐fold cross‐validation (1 × standard deviation (xstd) rule for pruning by cost‐complexity parameter).

### *In silico* simulations

Average time‐to‐survival profiles were simulated under a number of scenarios incorporating significant clinical covariates and the drug exposure if possible based on the final model.

## Results

Detailed demographics of the whole population have been described elsewhere.^3^ A total of 237 adult patients with a clinical diagnosis of TBM were enrolled in this PK substudy, but samples from two patients could not be analyzed due to limited volumes, and two patients died without blood samples for drug concentration measurements. These patients were excluded. Thus, 233 patients were included in the population PK/PD modeling analyses, where 118 and 115 patients were randomly assigned to standard treatment and intensified‐treatment groups, respectively. The baseline characteristics of the patients were balanced between the two treatment groups, with the exception of slightly higher bilirubin concentrations in the intensified arm (**Table** **S1**). Of the 233 patients included in the PD analysis, 38 patients (16 standard; 22 intensified) died within 9 months of follow‐up and 11 patients (5 standard; 6 intensified) died within 2 months.

### PK modeling

#### Rifampin

One thousand two hundred and forty‐nine plasma and 708 CSF samples were available for rifampin concentration measurements. Rifampin plasma concentration‐time profiles were best described by one disposition compartment with 5‐transit compartments characterizing the absorption process, along with an auto‐induction enzyme compartment and dose‐dependent bioavailability (**Figure** **S1**). The CSF concentration‐time profiles were accurately described by one CSF compartment linked to the plasma central compartment.

**Figure 1:**
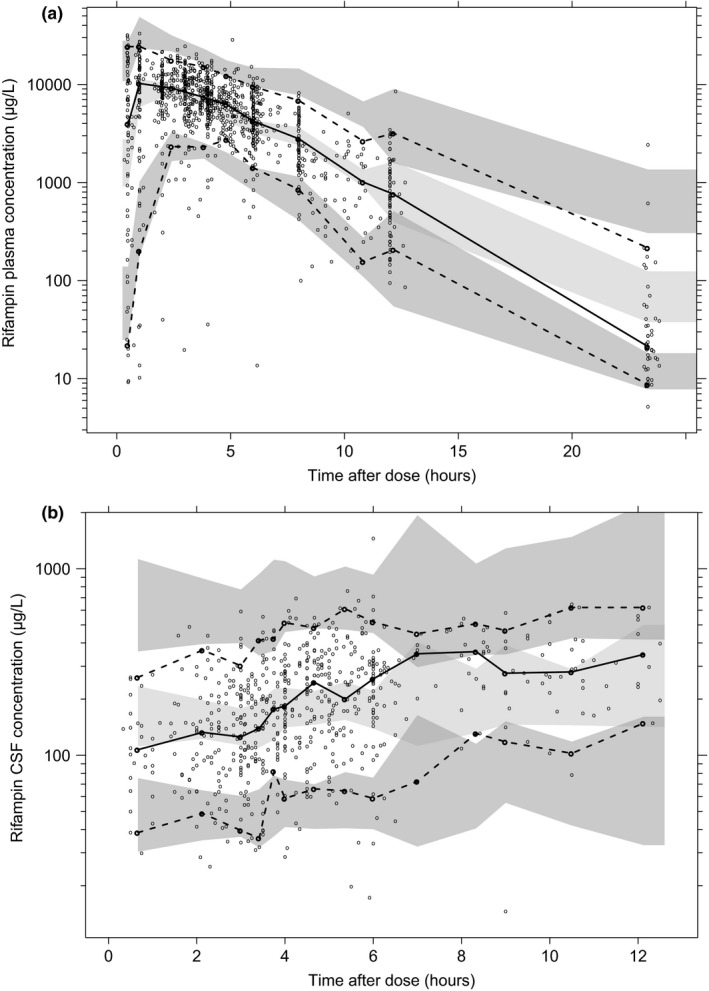
Prediction‐corrected and variability‐corrected visual predictive check of the final population pharmacokinetic model for (**a**) rifampin plasma and (**b**) CSF concentration based on 1,000 stochastic simulations. Open circles represent the observations, and solid lines represent the 5th, 50th, and 95th percentiles of the observed data. The shaded areas represent the 95% confidence intervals around the simulated 5th, 50th, and 95th percentiles. CSF, cerebrospinal fluid.

Allometric scaling by fat‐free mass performed better than total body weight. Inclusion of CSF protein concentration as a covariate on the blood‐brain penetration parameter (partition coefficient (PC)) showed a significant improvement in model fit (∆OFV = −17.486, *P* < 0.001), with 13.9% increase in PC per 1 g/L CSF protein. Other covariates did not have a significant impact on the PK properties of rifampin. Fifty‐three observations (2.8%; 44 from plasma, 9 from CSF) were identified as outliers. The visual predictive checks (VPC) and goodness‐of‐fit (GOF) diagnostic plots are shown in **Figures** 1 and **S2**, demonstrating good description of observed data and adequate predictive performance of the final model.

#### Isoniazid

**Figure 2:**
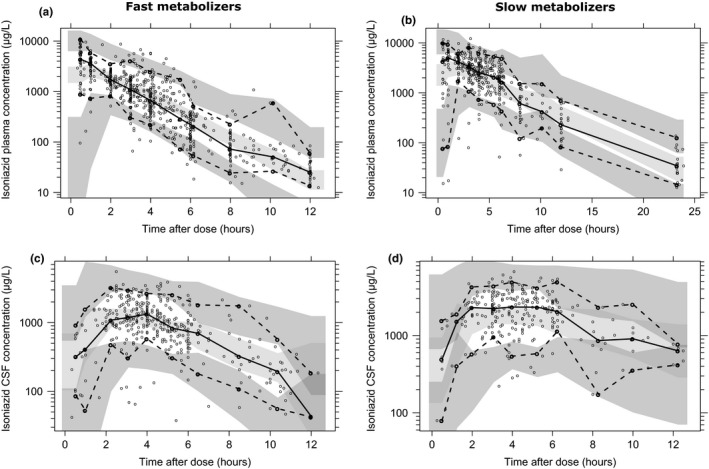
Visual predictive check of the final population pharmacokinetic model for isoniazid plasma and CSF concentration in (**a, c**) fast and (**b, d**) slow metabolizers based on 1,000 stochastic simulations. Open circles represent the observations, and solid lines represent the 5th, 50th, and 95th percentiles of the observed data. The shaded areas represent the 95% confidence intervals around the simulated 5th, 50th, and 95th percentiles. CSF, cerebrospinal fluid.

One thousand two hundred forty‐nine plasma and 708 CSF samples were available for isoniazid concentration measurements. Isoniazid plasma concentration‐time profiles were best described by one disposition compartment with five‐transit compartments in the absorption phase and first‐order elimination. The CSF concentration‐time profiles were accurately described by one CSF compartment linked to the plasma central compartment. Allometric scaling by fat‐free mass was implemented as it performed better than total body weight. Inclusion of a mixture model on elimination clearance showed a significant improvement in model fit (∆OFV = −27.54, *P* < 0.001). The PC parameter was estimated to 0.955 (i.e., close to 1) and therefore fixed to 1 without resulting in a significantly worse model fit (ΔOFV = 1.099). Thirty‐six observations (2.0%; 20 from plasma, 16 from CSF) were identified as outliers. No remarkable trends were seen in GOF diagnostic plots (**Figure** **S3**), and the VPC stratified by clearance phenotype showed good agreement between the observed data and model prediction (**Figure** 2).

#### Levofloxacin

Five hundred plasma and 217 CSF samples were available for levofloxacin concentration measurements. Levofloxacin plasma concentration‐time profiles were best described by one disposition compartment with two‐transit compartments in the absorption phase and first‐order elimination, and the CSF concentration‐time profiles were accurately characterized by a CSF compartment linked to the plasma central compartment. Allometric scaling by fat‐free mass was implemented as it performed similar to total body weight. Inclusion of creatinine clearance as a linear covariate on elimination clearance showed a significant improvement in model fit (∆OFV = −23.038, *P* < 0.001). Fifteen observations (2.1%; 11 from plasma, 4 from CSF) were identified as outliers. The GOF plot (**Figure** **S4**) and VPC (**Figure** **S5**) showed the good description and predictive performance of final model.

#### Ethambutol

Five hundred eighty‐four plasma samples from 131 patients were available for concentration measurements. Ethambutol plasma concentration‐time profiles were best described by two disposition compartments with one‐transit compartment in the absorption phase and first‐order elimination. Allometric scaling by fat‐free mass was implemented as it performed similar to total body weight. The collected covariates in this study did not have a significant impact on the PK properties of ethambutol. Ten (1.7%) plasma observations were identified as outliers. The GOF and VPC plots are presented in **Figures** **S6** and **S7**.

#### Pyrazinamide

One thousand sixty‐three plasma samples were available for concentration measurements. Pyrazinamide plasma concentration‐time profiles were best described by one disposition compartment with one‐transit compartment in the absorption phase and first‐order elimination. Allometric scaling by fat‐free mass was implemented as it performed similar to total body weight. Plasma AST was found to have a significant impact on elimination clearance using an exponential function (∆OFV = −30.31, *P* < 0.001). Twenty‐nine (2.8%) plasma observations were identified as outliers. No obvious trends were shown in GOF plots (**Figure** **S6**). The final model slightly overestimated the variability of data, as seen in the VPC plot (**Figure** **S7**).

#### Final PK parameter estimates

The final parameter estimates showed good precision with relatively small standard errors (**Tables** 1 and 2 for rifampin and isoniazid; **Tables** S2–S4 for levofloxacin, ethambutol, and pyrazinamide). The secondary parameters (i.e., C_max_ and AUC) derived from Empirical Bayes estimates from the final model are also presented.

**Table 1:** Final parameter estimates of rifampin population pharmacokinetics in patients with TBM

Parameter	NONMEM estimates (%RSE)	SIR median (95% CI)	CV for IIV (%RSE)	SIR median (95% CI)	Shrinkage (%)
*F* (%)	100 *fix*	—	33.2 (7.8)	—	31.6
MTT (hour)	0.99 (7.2)	0.98 (0.87–1.11)	68.8 (8.8)	68.8 (60.3–80.0)	27.4
No. transit comp.	5 *fix*	—	—	—	—
CL/F (L/hour)	10.1 (5.0)	10.0 (9.3–10.8)	33.9 (7.3)	34.0 (28.3–39.9)	32.4
*V*/*F* (L)	76.0 (4.9)	76.0 (70.2–82.0)	21.5 (27.1)	21.0 (10.0–30.3)	59.1
E_max_	1.16 *fix*	—	—	—	—
EC_50_ (mg/L)	0.0699 *fix*	—	—	—	—
*k*_enz_ (1/hour)	0.00603 *fix*	—	—	—	—
F_max_	0.504 *fix*	—	—	—	—
ED_50_ (mg)	67 *fix*	—	—	—	—
*Q*/*F* (L/hour)	0.00387 (14.5)	0.00393 (0.00283–0.00484)	96.1 (8.5)	96.6 (80.9–115.3)	46.0
*V*_csf_ (L)	0.15 *fix*	–	—	—	—
PC	0.0712 (4.3)	0.0709 (0.0649–0.0764)	—	—	—
RUV for plasma	0.253 (3.3)	0.254 (0.230–0.278)	—	—	14.4
RUV for CSF	0.490 (5.4)	0.488 (0.434–0.546)	—	—	10.8
Covariate relationships					
CSF protein on PC (%)	14.1 (20.6)	13.8 (9.1–19.3)	—	—	—

Secondary parameters	Standard therapy	Intensified therapy	All patients		
Day 14 C_max plasma_ (mg/L)	10.6 (2.8–21.6)	18.2 (0.9–41.8)	12.9 (0.9–41.8)		
Day 14 AUC_plasma_ (hour·mg/L)	48.2 (18.2–93.8)	82.5 (8.7–161.0)	64.0 (8.7–161.0)		
Day 14 C_max CSF_ (μg/L)	189.3 (64.9–566.6)	330.8 (35.1–828.8)	255.9 (35.1–828.8)		
Day 14 AUC_CSF_ (hour·mg/L)	3.5 (1.2–9.6)	6.0 (0.7–15.1)	4.8 (0.7–15.1)		

Population estimates in the table are given for a “typical” patient with free fat mass of 70 kg. Coefficients of variation for interindividual variability (IIV) were calculated as 100 × (e^variance^)^1/2^. Relative standard errors (%RSE) were calculated as 100 × (standard deviation/mean). Secondary‐parameter estimates were calculated from the Empirical Bayes *post hoc* estimates and presented as median (range).

F_max_ and ED_50_ were implemented on *F* using an E_max_‐like function $\left(F=\left(1+\frac{F_{\max}\cdot \left(\text{Dose}-450\right)}{{\text{ED}}_{50}+\left(\text{Dose}-450\right)}\right)\right)$). CSF protein was included on parameter PC using a linear model $\left(\text{PC}={\text{PC}}_{\text{TV}}\cdot \left(1+\theta \cdot \left({\text{protein}}_{\text{CSF}}-1.6\right)\right)\right)$, and ${\text{PC}}_{\text{TV}}$ is the typical PC value of the population.

AUC, area under the concentration‐time curve; CL/F, elimination clearance; C_max_, peak concentration; CSF, cerebrospinal fluid; *F*, relative bioavailability; EC_50_, plasma concentration corresponding to 50% of E_max_; ED_50_, dose corresponding to half the F_max_; E_max_, maximum increase in enzyme formation rate; F_max_, maximum increase in relative bioavailability; *k*_enz_, enzyme degradation rate; MTT, mean transit time; No. transit comp., number of transit compartments in the absorption phase; PC, partition coefficient between central and CSF compartment; *Q*/*F*, intercompartmental clearance; RUV, additive residue error on log scale; SIR, sampling importance resampling; TBM, tuberculous meningitis; V/F, central volume of distribution; V_CSF_/F, CSF volume of distribution.

**Table 2:** Final parameter estimates of isoniazid population pharmacokinetics in patients with TBM

Parameter	NONMEM estimates (%RSE)	SIR median (95%CI)	CV for IIV (%RSE)	SIR median (95%CI)	Shrinkage (%)
*F* (%)	100 *fix*	–	46.0 (4.3)	46.1 (40.5–51.5)	32.7
MTT (hour)	0.357 (15.6)	0.362 (0.329–0.426)	102.5 (11.9)	101.6 (84.9–118.8)	42.2
No. transit comp.	5 *fix*	–	–	–	
CL_slow_/F (L/hour)	18.1 (5.9)	18.1 (16.3–19.8)	14.7 (15.4)	14.7 (11.4–18.7)	41.3
CL_fast_/F (L/hour)	40.7 (2.4)	40.6 (37.3–44.0)			
Probability of slow metabolizer	0.399 (11.3)	–	–	–	–
*V*/*F* (L)	96.7 (5.7)	–	–	–	–
*Q*/*F* (L/hour)	0.0344 (9.7)	0.0345 (0.0298–0.0351)	71.8 (6.5)	71.7 (60.0–82.6)	47.3
*V*_csf_ (L)	0.15 *fix*	–	–	–	–
PC	1 *fix*	–	–	–	–
RUV for plasma	0.299 (4.7)	0.300 (0.271–0.326)	–	–	9.7
RUV for CSF	0.274 (16.6)	0.274 (0.240–0.309)	–	–	16.6

Secondary parameters	Slow metabolizer	Fast metabolizer	All patients		
Day 14 C_max plasma_ (mg/L)	4.72 (0.30–7.12)	3.56 (0.77–6.96)	3.91 (0.30–7.12)		
Day 14 C_max CSF_ (mg/L)	2.51 (0.17–4.45)	1.40 (0.15–4.64)	1.80 (0.15–4.64)		
Day 14 AUC_CSF_ or AUC_plasma_ (hour·mg/L)	24.2 (1.5–41.9)	8.9 (1.5–19.0)	11.6 (1.5–41.9)		

Population estimates in the table are given for a “typical” patients with free fat mass of 70 kg. The calculation of IIV, RSE, as well as the secondary parameters refer to **Table** 1. The AUC_CSF_ was equal to AUC_plasma_, as the PC parameter was 1 in the model.

AUC, area under the concentration‐time curve; CL_slow_ /F, elimination clearance for slow metabolizer; CL_fast_/*F*, elimination clearance for fast metabolizer; *F*, relative bioavailability; IIV, interindividual variability; MTT, mean transit time; No. transit comp., number of transit compartments in the absorption phase; *Q*/*F*, inter‐compartmental clearance; RSE, relative standard errors; RUV,  additive residue error on log scale; SIR, sampling importance resampling; TBM, tuberculous meningitis; *V*/*F*, central volume of distribution; V_CSF_/F, CSF volume of distribution.

Rifampin 15 mg/kg resulted in increased drug exposures in plasma and CSF compared with 10 mg/kg (**Table** 1): Day 14 AUC rose from 48.2 hour·mg/L (range 18.2–93.8) to 82.5 hour·mg/L (range 8.7–161.0) in plasma and from 3.5 hour·mg/L (range 1.2–9.6) to 6.0 hour·mg/L (range 0.7–15.1) in CSF. We also used the model to predict exposure after 2 days (before substantial metabolism induction) and after 9 days, which is a timepoint often presented in previous studies.^5^ The model predicted plasma C_max_ and AUC at day 2 were 20.5 (range 1.3–45.0) mg/L and 131.2 (range 12.9–268.2) mg·hour/L, respectively, at 15 mg/kg/day. The model predicted plasma AUC at day 9 of treatment were 51.1 (range 19.2–101.0) and 92.9 (range 9.3–176.0) mg·hour/L for 10 and 15 mg/kg daily dose, respectively.

Isoniazid exposures had a bimodal distribution with fast and slow metabolizers having substantially different plasma and CSF exposure (**Table** 2). Fast metabolizers had plasma and CSF AUC of 8.9 hour·mg/L (range 1.5–19.0), compared with 24.2 hour·mg/L (range 1.5–41.9) for slow metabolizers.

The estimated median (range) CSF C_max_/MIC for rifampin, isoniazid, and levofloxacin were 1.4 (0.19–4.9), 36.0 (2.9–92.7) and 3.7 (1.2–7.0) with a reported MIC value of 0.25, 0.05, and 1.0 mg/L, respectively.^17^

#### PD modeling

HIV coinfection, bodyweight, GCS, albumin, AST, ALT, and MRC grade were associated with death (*P* < 0.01) by univariate analysis (**Table** **S5**); with correlations (*r* > 0.69) between AST and ALT, GCS, and MRC. Given their clinical importance, GCS, AST, HIV coinfection, bodyweight, and albumin were investigated further in time‐to‐event model analyses.

**Table** 3 shows the exposure of each antituberculosis drug at steady state (day 14 after the initiation of treatment) in those who died or survived. Neither rifampin plasma nor CSF exposure (C_max_ and AUC) was significantly associated with survival or death. In contrast, lower isoniazid CSF C_max_ and AUC was significantly associated with death (*P* < 0.01). The exposures to levofloxacin, ethambutol, and pyrazinamide in plasma and/or CSF showed no associations with outcome. The plasma ethambutol exposure was derived from 131/233 patients, and therefore not investigated further in time‐to‐event modeling.

**Table 3:** The exposure to each antituberculosis drug at steady state (day 14 after the treatment) in those who survived and died from TBM

	Survival			Death			*P* value
	Standard arm (*n* = 102)	Intensified arm (*n* = 93)	Subtotal (*n* = 195)	Standard arm (*n* = 16)	Intensified arm (*n* = 22)	Subtotal (*n* = 38)	
Rifampin							
C_max plasma_ (mg/L)	10.7 (3.6–21.6)	18.6 (3.6–41.8)	12.7 (3.6–41.8)	9.5 (2.8–16.2)	17.8 (0.9–26.5)	14.8 (0.9–26.5)	0.808
AUC_plasma_ (hour·mg/L)	49.7 (19.2–93.8)	82.9 (15.0–159.6)	62.2 (15.0–159.6)	42.5 (18.2–79.6)	81.0 (8.7–161.0)	73.6 (8.7–161.0)	0.901
C_max CSF_ (μg/L)	192.9 (64.9–566.6)	338.3 (55.0–828.8)	252.9 (55.0–828.8)	174.9 (81.9–485.7)	300.3 (35.1–529.0)	265.2 (35.1–529.0)	0.551
AUC_CSF_ (hour·mg/L)	3.5 (1.3–7.6)	6.0 (1.0–15.2)	4.6 (1.0–15.1)	3.2 (1.2–9.6)	5.9 (0.7–9.1)	5.1 (0.7–9.6)	0.725
Isoniazid							
C_max plasma_ (mg/L)	4.37 (1.22–7.02)	3.7 (0.77–7.12)	4.04 (0.77–7.12)	3.42 (1.78–5.65)	3.25 (0.30–4.97)	3.28 (0.30–5.65)	< 0.001
C_max CSF_ (mg/L)	2.11 (0.40–4.45)	1.73 (0.15–4.64)	1.96 (0.15–4.64)	1.49 (0.65–3.22)	1.36 (0.17–2.49)	1.38 (0.17–3.22)	< 0.001
AUC_Plasma or CSF_ (hour·mg/L)	13.6 (3.2–40.2)	11.0 (1.5–41.9)	12.0 (1.5–41.9)	10.4 (4.0–30.3)	8.3 (1.5–26.2)	8.4 (1.5–30.3)	0.001
Levofloxacin							
C_max plasma_ (mg/L)	–	8.9 (2.5–16.7)	–	–	8.9 (3.4–16.2)	–	0.664^a^
AUC_plasma_ (hour·mg/L)	–	69.9 (24.1–137.2)	–	–	70.3 (31.9–124.3)	–	0.506^a^
C_max CSF_ (mg/L)	–	3.6 (1.2–7.0)	–	–	3.6 (1.6–6.5)	–	0.153^a^
AUC_CSF_ (hour·mg/L)	–	39.5 (13.6–77.7)	–	–	39.8 (18.1–70.4)	–	0.461^a^
Ethambutol							
C_max plasma_ (mg/L)	2.6 (1.4–5.0)	2.3 (1.3–5.4)	2.4 (1.3–5.4)	2.1 (1.1–2.8)	2.5 (2.0–3.3)	2.4 (1.1–3.3)	0.663
AUC_plasma_ (hour·mg/L)	16.3 (11.3–24.7)	15.6 (10.7–25.6)	16.2 (10.7–25.6)	15.0 (11.8–24.4)	15.5 (12.6–20.4)	15.3 (11.8–24.4)	0.379
Pyrazinamide							
C_max plasma_ (mg/L)	42.1 (4.9–106.9)	40.6 (11.6–96.0)	41.8 (4.9–106.9)	35.4 (28.9–71.0)	38.0 (9.8–67.8)	37.6 (9.8–71.0)	0.190
AUC_plasma_ (hour·mg/L)	378.9 (73.3–766.3)	379.1 (139.7–1,349.2)	379.1 (73.3–1,349.2)	366.1 (214.9–717.6)	385.1 (106.0–1,029.0)	372.4 (106.0–1,029.0)	0.241

The data were reported as median (range). One‐way analysis of variance was performed to compare the difference between survival and death patients.

AUC, area under the concentration‐time curve; C_max_, peak concentration; TBM, tuberculous meningitis.

a Comparison between intensified survival and death patients. Ethambutol exposure were derived from 107 survival patients (53 for standard and 54 for intensified arm) and 24 deaths (10 for standard and 14 for intensified arm). The isoniazid AUC_CSF_ was equal to AUC_plasma_, as assuming 100% penetrating from plasma to CSF (cerebrospinal fluid). The AUC and C_max_ of levofloxacin were not reported for the patients without levofloxacin treatment in standard treatment arm.

The best base hazard model for time‐to‐death was a log‐normally distributed model, defined by median of distribution (μ) and standard deviation (σ). Inclusion GCS on μ improved model fit (∆OFV = −31.305, *P* < 0.001). Inclusion of either HIV coinfection, or albumin, or AST on μ improved model fit further (∆OFV = −11.568, −12.553 and −17.973, *P* < 0.001). Considering the correlation between these three clinical covariates, and the importance of HIV coinfection to outcome, we retained HIV coinfection as a covariate in the model. Furthermore, inclusion of bodyweight did not improve the model fit (∆OFV = −4.27, *P* > 0.01).

Inclusion of individual rifampin exposures (plasma and/or CSF day 14 C_max_ and AUC) did not significantly affect the hazard (*P* > 0.05), using either a linear, E_max_ or sigmoidal E_max_ model. In contrast, individual isoniazid exposures (both day 14 CSF C_max_ and AUC) were found to significantly affect the hazard, with a higher exposure leading to a decreased probability of death. The model which included CSF C_max_ performed slightly better than CSF AUC (∆OFV = −0.88). A sigmoidal E_max_ relationship described the data better than a linear and E_max_ relationship (∆OFV = −5.74 and −4.72, respectively). The estimated EC_50_ for CSF C_max_ and AUC were 1.37 mg/L and 7.03 mg·hour/L, respectively. No significant effect of levofloxacin and pyrazinamide exposure on hazard was detected when investigated alone or together with isoniazid exposure.

The parameter estimates of final PD model, presented in **Table** 4, showed good precision with relatively small standard errors. The visual predictive checks illustrated good description of observed data and adequate predictive performance of the final model (**Figure** **S8**).

**Table 4:** Final parameter estimates of the time‐to‐event model describing the time to death from TBM

Parameter	NONMEM estimates (%RSE)	SIR median (95%CI)
Using isoniazid C_max_ linked to hazard of death		
μ	3.42 (18.1)	3.48 (2.37–4.54)
σ	1.18 (16.4)	1.22 (0.86–1.64)
Glasgow coma scale on μ (%)	9.07 (14.9)	8.83 (6.36–12.68)
HIV coinfection on μ (%)	–27.7 (35.3)	–27.4 (−54.5 to −11.1)
IC_50_ (mg/L)	1.37 (34.3)	1.43 (0.54–2.44)
γ	2.79 (30.3)	2.77 (1.28–5.81)
Using isoniazid AUC linked to hazard of death		
μ	3.29 (18.4)	3.33 (2.20–4.47)
σ	1.12 (16.2)	1.14 (0.79–1.57)
Glasgow coma scale on μ (%)	9.39 (13.6)	9.34 (6.34–13.03)
HIV coinfection on μ (%)	–33.5 (29.6)	–33.6 (−50.9 to −14.9)
IC_50_ (hour·mg/L)	7.03 (45.1)	6.99 (2.34–14.34)
γ	1.74 (28.1)	1.70 (0.87–3.08)

The hazard function can be described by the equations below.

$h\left(t\right)=h_{0}\left(t\right)\times \left(1-\frac{C_{\text{max,INH}}^{\gamma }}{C_{\text{max,INH}}^{\gamma }+IC_{50,\text{INH}}^{\gamma }}\right)$, $h_{0}\left(t\right)=\frac{{\left(\sigma t\sqrt{2\pi }\right)}^{-1}e^{\left(-\frac{1}{2}Z^{2}\right)}}{1-\Phi (Z)}$,$Z=\frac{\ln\left(t\right)-\mu }{\sigma }$

where *t* represents the survival time, *h*(*t*) is the hazard function. *h*_0_(*t*) is the baseline hazard followed lognormal distribution, where μ and σ are the median and standard deviation of the distribution. IC_50_ is 50% inhibitory C_max_ or AUC. γ is the slope‐factor for the drug effect. Glasgow coma scale (GCS) was included on parameter μ using a linear model, and HIV coinfection was implemented on μ using a proportional model $\left(\mu =\mu_{\text{TV}}\cdot \left(1+\theta_{\text{GCS}}\cdot \left(\text{GCS}-14\right)\right)\cdot \left(1+\theta_{\text{HIV}}\cdot \text{HIV}\right)\right)$, where $\mu_{\text{TV}}$ was the typical value of the parameter. The isoniazid exposure was included on the hazard.

AUC, area under the concentration‐time curve; C_max_, peak concentration; RSE, relative standard errors; SIR, sampling importance resampling.

The significant PD covariates identified by population modeling (GCS, HIV coinfection, and isoniazid CSF exposure), as well as the rifampin and levofloxacin exposures were evaluated in the CART analysis. The final full CART tree included four terminal nodes: GCS, HIV coinfection, isoniazid CSF C_max_, and rifampin CSF C_max_ (**Figure** **S9** **a**). However, higher rifampin exposures led to the prediction of worse outcomes and were therefore discarded as clinically implausible (**Figure** **S9** **b**). The variable with the greatest discriminative power was GCS, followed by isoniazid CSF C_max_ and HIV coinfection. Isoniazid CSF AUC was not included in the model under CART analysis.

We performed a sensitivity analysis by excluding deaths occurring in the first 2 months and found consistent results that GCS, HIV coinfection, and isoniazid CSF C_max_ were significantly related to outcome and that rifampin exposures were not relevant to the hazard of death.

### Simulations under clinical scenarios

**Figure 3:**
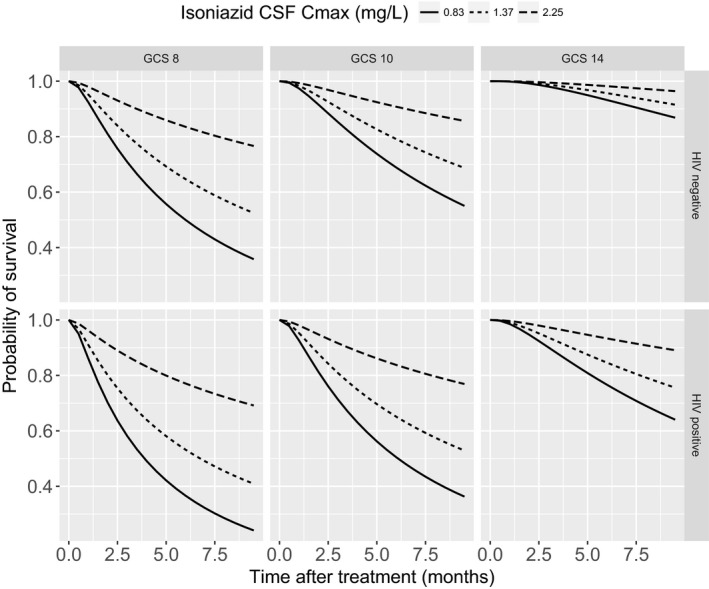
Predicted Kaplan‐Meier curves for patients according to HIV, Glasgow coma scale (GCS), and isoniazid exposure.

The GCS, HIV coinfection as well as the isoniazid CSF C_max_ levels (0.83, 1.37, and 2.25 mg/L, corresponding to EC_20_, EC_50_, and EC_80_) were considered in the simulation scenarios. The probability of survival was increased along with increased isoniazid exposure and GCS, and decreased with HIV coinfection, as demonstrated in **Figure** 3, showing the average predicted time to survival profiles with different isoniazid CSF C_max_ levels.

## Discussion

To our best knowledge, this is the first study to investigate dose‐exposure‐response relationships of five antituberculosis drugs in patients with TBM in a large clinical trial using a PK/PD modeling approach. Our results did not support a dose–response relationship of rifampin in the study population. By contrast, we found that higher isoniazid exposure was related to reducing the hazard of death within 9 months of treatment, after adjustment for the effects of GCS and HIV coinfection.

In this study, the rifampin PK characteristics were comparable to previous reports in patients with TBM, with exposure increasing with dose. Many previous studies have reported exposures (C_max_ and AUC) after 2 and 9 days of treatment. The model developed here predicted an increase in plasma C_max_ from 11.9 mg/L (10 mg/kg) to 20.5 mg/L (15 mg/kg), and plasma AUC from 74.6 mg·hour/L (10 mg/kg) to 131.2 mg·hour/L (15 mg/kg), when measured at day 2. These values are similar to those reported by Dian *et al*. (C_max_ 7.2 mg/L, AUC 53.5 mg·hour/L with 10 mg/kg)^5^ and Yunivita *et al*. using rifampin 17 mg/kg (C_max_ 14.3 mg/L and AUC 131.4 mg·hour/L).^8^ The day 9 predicted estimates for AUC and C_max_ were also similar to the values reported by Dian and Yunvita, indicating that the rifampin dose used in our trial had the predicted increase in exposure.

Evidence has accumulated that higher rifampin exposure is associated with increased survival from TBM.^5^, ^6^, ^7^, ^8^ A recent study suggested that exceeding a threshold value of plasma AUC_0–6_ of 70 mg·hour/L (AUC_0–24_ 116 mg·hour/L) and a C_max_ of 22 mg/L in the first 3 days of rifampin administration is associated with increased survival.^7^ In our trial, the thresholds were not met for C_max_ or AUC in 99.2% (117/118) and 90.7% (107/118) of patients given 10 mg/kg rifampin. At 15 mg/kg, however, the thresholds were met for C_max_ in 64.3% (74/115) and 69.9% (80/115) of patients.

These findings might explain the lack of effect of higher rifampin doses on outcome in the trial. However, the lack of any relationship between rifampin exposure and outcome is surprising given the previous data from Indonesia. It is possible that the relatively low exposures achieved, even with 15 mg/kg rifampin, and the limited range of AUC reduced the study’s power to demonstrate a significant relationship. However, exposures at 15 mg/kg were similar to those achieved with 13 mg/kg given intravenously in the Indonesian study, which reported a significant association with survival.

Isoniazid is an important part of the first line treatment for TBM due to high early bactericidal activity within the first 2 days of treatment^18^ and its excellent CSF penetration.^7^, ^19^, ^20^ The CSF‐to‐plasma free concentration ratio (AUC_total,CSF_/AUC_unbounded,plasma_) was estimated to be close to 1 (1.11) in the current study using a reported plasma protein binding of 14%.^21^ Isoniazid is metabolized by the genetically polymorphic NAT2, with fast and slow metabolizer phenotypes.^12^ The PK characteristics of isoniazid in our study were similar to those reported in pulmonary tuberculosis,^22^ with median AUC for slow and fast metabolizers of 24.2 and 8.9 hour·mg/L, respectively (previously reported values of 17.1 and 9.9 hour·mg/L). Low isoniazid exposure has been associated with poor treatment outcomes in pulmonary tuberculosis,^23^, ^24^ but there are few data from patients with TBM. Our PD modeling, using either parametric survival analysis or CART approach, suggested that isoniazid exposure was associated with survival. This was supported further by a sensitivity analysis, excluding deaths occurring in the first 2 months, estimating a somewhat lower EC_50_ value for isoniazid in this subanalysis (i.e., 1.17 vs. 1.37 mg/L). This finding suggests that higher exposures to isoniazid might be needed in the first 2 months of treatment. Twenty‐eight of the 38 patients who died were fast metabolizers with lower isoniazid exposures. Increasing the dose of isoniazid for fast metabolizers, e.g., to 10 mg/kg per day would reach the 80% inhibitory isoniazid CSF C_max_ (2.25 mg/L) for the hazard. Moreover, the isoniazid CSF C_max_ was comparable between patients with resistant (*n* = 34, 1.79 (range 0.40–3.79) mg/L) and sensitive (*n* = 111, 1.73 (range 0.15–4.64) mg/L) infections. Again, the isoniazid resistance rate was 23.4% (34/145) in the study population and was evenly balanced between patients that died and survived (15.4% (4/26) vs. 25.2% (30/119), *P* = 0.415). This suggests that the relatively high isoniazid resistance rate observed did not have a clinically significant impact on the outcome, under the current anti‐TBM dosage regimen. Taken together, our data suggest that acetylator status may have an important impact on outcome from TBM and isoniazid doses ≥ 10 mg/kg/day should be investigated in adults, especially fast acetylators.

Our data confirm good CSF penetration of levofloxacin,^25^ resulting in a CSF‐to‐plasma free concentration ratio of 0.76, using a reported plasma protein binding of 25%,^21^ but we did not observe an exposure–response relationship with levofloxacin. Previous studies have suggested a U‐shaped exposure–response relationship with fluoroquinolones in TBM treatment,^26^ and a study in children suggested higher pyrazinamide exposures were linked to better outcomes.^27^ Conversely, however, higher pyrazinamide CSF exposures have been associated with increased mortality and neurological toxicity in HIV‐associated TBM.^28^ Of note, we did not find that HIV coinfection influenced exposures of any of the antituberculosis drugs, contrary to some previous reports.^29^, ^30^, ^31^

Our study has several limitations. First, we were unable to sample the entire trial population, which may have reduced our power to define exposure–response relationships. Second, the trial only explored rifampin doses up to 15 mg/kg, which would now not be considered high against emerging data on up to 40 mg/kg and therefore did not allow analysis of much higher exposures on outcome. Third, only 144/233 (61.8%) had *Mycobacterium tuberculosis* isolated from CSF, which meant the relationship between MIC, exposure, and outcome could only be explored in a subset. The substantial proportion of patients treated for TBM on clinical grounds alone also introduces the possibility of alternative causes of meningitis, which would alter drug exposure responses. Moreover, the limited data set (i.e., relatively small number of deaths) probably reduced the power to identify the influential covariates. Fourth, lumbar puncture is an invasive procedure and multiple, serial CSF sampling is not possible. As a consequence, we have limited data on drug exposures at the site of disease and, like other investigators, we have had to extrapolate exposure from plasma drug concentrations. This approach has limitations given the variable and sometimes restricted passage of the antituberculosis drugs across the blood‐brain barrier. In addition, considering the high plasma protein binding of 88%,^21^ the rifampin free concentration in CSF might be more relevant to the outcome and more suitable to evaluate the CSF penetration in the TBM patients. However, we did not measure the protein binding in CSF in this study. The CSF total concentration‐to‐plasma free concentration ratio was estimated to be 0.59 in this study. Fifth, CSF is often not a good surrogate for drug concentrations in brain extracellular fluid,^32^, ^33^ but extracellular fluid samples were not collected in this study.

## Conclusion

Our clinical trial failed to show that the addition of higher dose rifampin (15 mg/kg/day) and levofloxacin (1,000 mg/day) to standard antituberculosis treatment for the first 2 months of therapy has any impact on clinical outcomes.^3^ The current study showed that 15 mg/kg/day rifampin increased plasma exposures substantially, with AUC similar to those associated with improvements in survival in other studies. However, we were unable to find any significant relationship between increased rifampin exposure and survival in our cohort. In contrast, we found that isoniazid exposure was associated with survival, with low exposure predictive of death and linked to the fast metabolizer phenotype. While phase III trials of high dose (> 30 mg/kg) rifampin for TBM remain justified, consideration should also be given to exploring higher doses of isoniazid for the treatment of adults with TBM, especially fast acetylators.

## Author Contributions

J.D., G.T., N.T.T.T., and J.T. wrote the manuscript; J.T., D.H., T.P., and G.T. designed the research; D.H., C.T.H.T., M.T.H.N., P.H.N., C.V.V.N., and L.P.P. performed the research; J.D. and J.T. analyzed the data; T.P. and T.V.P. contributed new reagents/analytical tools.

## Funding

The study was funded by the Wellcome Trust, United Kingdom. Clinical trial registration: ISRCTN61649292. The work of the Department of Clinical Pharmacology, Mahidol‐Oxford Tropical Medicine Research Unit is partly funded by the Wellcome Trust of Great Britain and the Bill & Melinda Gates Foundation. The funders had no part in the study design, implementation and analysis of the result, or the decision to publish this manuscript.

## Conflict of Interest

The authors declared no competing interests for this work.

## Supporting information


**Supplementary Text.** Supplementary Methods, Supplementary Results, Tables S1–S6, Figures S1–S9.

**NONMEM Code.**
