# Supplementary material for: Pharmacokinetics and Pharmacodynamics of Intensive Antituberculosis Treatment of Tuberculous Meningitis
Source: Clin Pharmacol Ther. 2020 Feb 29;107(4):1023–33. doi: 10.1002/cpt.1783 (PMC7158205; doi:10.1002/cpt.1783)
Supplement: Supplementary file 1 — Supplementary Text. Supplementary Methods, Supplementary Results, Tables S1–S6, Figures S1–S9. [file CPT-107-1023-s001.pdf]

SUPPLEMENTARY TEXT

Pharmacokinetics and pharmacodynamics of intensive antituberculosis treatment of tuberculous meningitis

SUPPLEMENTARY METHODS

Blood samples collection

Blood samples were collected in lithium heparin tubes and immediately (within 5 min) centrifuged at 2,000 g for 15 min. The plasma and CSF samples were carefully aliquoted into ascorbic acid pre-coated cryotubes for measurement of isoniazid, rifampin, pyrazinamide and ethambutol, or normal cryotubes for levofloxacin and stored at -20°C in the ward. Levofloxacin was measured in the Oxford University Clinical Research Unit (OUCRU) laboratory, Vietnam; all other drugs were measured in the Department of Clinical Pharmacology, Mahidol Oxford Tropical Medicine Research Unit, Thailand. Samples were stored at -80°C until analysis.

LC-MS/MS quantification of rifampin, isoniazid, pyrazinamide and ethambutol

Plasma and cerebrospinal fluid (CSF) concentrations of rifampin, isoniazid and plasma concentration of pyrazinamide and ethambutol were measured, using an LC-MS/MS based assay. Stock solutions of analytes (10 mg/mL) and stable isotope-labeled internal standards (SIL-IS) (1 mg/mL) were prepared in methanol (MeOH)/water (1:1 v/v) for pyrazinamide, isoniazid and ethambutol and in pure MeOH for rifampin. Sample preparation consisted of cold acidic protein precipitation (PPT) followed by a phospholipid removal performed on 96-well Phenomenex PHREE plates (Macclesfield, U.K.). One microliter was injected into the equilibrated LC/MS system.

The LC system was an Agilent 1290 system consisting of a binary LC pump, a vacuum degasser, a temperature-controlled micro-well plate auto-ampler set at 4°C and a temperature-controlled column compartment set at 40°C (Agilent technologies, CA, USA). Data acquisition and quantification were performed using Analyst 1.6.0 (Applied Biosystems/MDS Sciex, CA, USA). The 4 analytes and their internal standards were analyzed on a ZIC-cHILIC (5 µm, 50 × 2.1 mm, 3 mm, 100Å) column protected by a ZIC-cHILIC guard column (16 × 1.0 mm, 5 mm) (Merck Sequant, Umea, Sweden). The mobile phase consisted of solvent A (Acetonitrile-ammonium acetate 1 mM (97:3, v/v)) and solvent B (Acetonitrile-ammonium acetate 20 mM (80-20, v/v)). The mobile phase was delivered at 500 µL/min using a gradient system of 100%A to 100%B (0-5 min), followed by a double wash gradient (7-12 min) to avoid column carry-over for ethambutol.

An API 5000 triple quadrupole mass spectrometer (Applied Biosystems/MDS Sciex) with a TurboV ionization source (TIS) interface operating in the positive ion mode, was used for the multiple reactions monitoring LC-MS/MS analysis. Quantifications were performed using the following selected reaction monitoring: m/z 124.2 → 81.0 and 127.1 → 99.9 for pyrazinamide and SIL- pyrazinamide; m/z 138.1 → 121.1 and 142.2 → 125.0 for isoniazid and SIL- isoniazid; m/z 823.4 → 791.4 and 827.4 → 795.3 for rifampin and SIL- rifampin and m/z 205.3 → 116.1 and 209.3 → 120.1 for ethambutol and SIL- ethambutol. The measurable ranges (lower limit of quantification (LLOQ) to upper limit of quantification (ULOQ)) in plasma were 8.00 to 18,400 ng/mL for rifampin, 800 to 57,500 ng/mL for pyrazinamide, 12.0 to 13,800

ng/mL for isoniazid and 8.00 to 4,830 ng/mL for ethambutol. Precisions and accuracies of quality control (QC) samples during the analysis of plasma clinical samples are shown in Table S6.

The simultaneous quantification of the 4 analytes in human CSF was challenged by the low concentrations, a significant matrix effect, and coeluting endogenous interferences. For those reasons, only rifampin and isoniazid were quantified in CSF. The CSF samples were processed following the method described above for plasma, but with one additional step before the protein precipitation: 20 mL of a Bovine Serum Albumin (BSA) solution (at 200 mg/mL in MS grade water) was added to 100 mL of CSF samples. The measurable ranges (LLOQ to ULOQ) in CSF were 36.0 to 4,600 ng/mL for isoniazid and 32.0 to 1,600 ng/mL for rifampin. Precisions and accuracies of QC samples during the analysis of CSF clinical samples are shown in Table S6.

HPLC analysis of levofloxacin

Levofloxacin concentrations in plasma and CSF were measured by high-performance liquid chromatography (HPLC) with time-programmed fluorescence detection after solid-phase extraction according to in-situ published method (1). The LLOQ of both plasma and CSF samples were 20 ng/mL. Total precision of all quality control plasma and CSF samples (low, middle and high concentrations) was <5.21%.

PK/PD modelling

The population PK analysis was performed using nonlinear mixed-effects modelling in the software NONMEM (version 7.4, ICON Development Solutions, Ellicott City, MD, USA), compiled using gFortran (version 4.60). Perl-speaks-NONMEM (PsN; version 4.6.0) and R (version 3.2.0, http://www.r-project.org/) were used to evaluate the goodness of fit and output visualizations. The first-order conditional estimation method including η-ε interactions (FOCE-I) and the Laplace algorithm were used throughout the population PK and PD model-building procedure, respectively. Discrimination between models during the model building phase was based on standard visual diagnostics and the objective function value (OFV), calculated as proportional to twice the log-likelihood of the data. A reduction in OFV (ΔOFV) of 3.84 and 6.64 was considered a significant improvement at $p < 0.05$ and $p < 0.01$, respectively, between two hierarchical models after inclusion of one additional parameter (one degree of freedom difference).

PK modelling

If the proportion of samples below the LLOQ was low (<5%), these LLOQ samples were omitted from the model development process (M1 approach) and not considered further (2). If the proportion of LLOQ samples was non-negligible (>5%), LLOQ concentration-data were evaluated by comparing the proportion of predicted and observed concentrations below the LLOQ, using categorical visual predictive checks. If model misspecification was present when omitting LLOQ data, the M6 (imputing the first concentration below LLOQ within a patient as half of the LLOQ) and M3 (maximizing the likelihood to predict censored data) methods were evaluated.

Different disposition models, including one- and two-compartment disposition models, were tested for each drug. A flexible transit-absorption model, with a stepwise addition of a fixed number of 1-10 transit-compartments, was employed to describe the absorption process. One CSF compartment was added in the models (when CSF concentration was available) to describe the transfer process between the plasma

and CSF, with a parameter PC (partition coefficient) to quantify the transfer between the central and CSF compartments. Inter-individual variability was added exponentially to all parameters, and assumed to be normally distributed with a zero mean and variance ω^2. Relative bioavailability (F) was fixed to unity in the population, allowing quantification of the inter-individual variability in the absorption process. The residual unexplained variability, assumed to be normally distributed with a zero mean and variance σ^2, was modelled as an additive error on log-transformed concentrations, which is approximately equivalent to an exponential residual error on an arithmetic scale. Simulation/evaluation-based diagnostics was used to identify concentration-time data outliers (simeval command in PsN) within each drug model. The final population PK models were re-estimated after removal of these outliers.

Model evaluation

Basic goodness-of-fit diagnostics were used to evaluate systematic errors and model misspecification for all population PK models. The sampling importance resampling (SIR) approach (3) was used to calculate parameter uncertainty in the final models (samples = 2,000, resamples = 1,000). The overall predictive performance of the final models were evaluated using simulation-based diagnostics (i.e. prediction-corrected visual predictive checks, n = 2,000 simulations).

SUPPLEMENTARY RESULTS

Handling of data below the LLOQ

For rifampin, 42/1,249 (3.4%) of plasma samples and 55/708 (7.8%) of CSF samples were below the LLOQ. M1 and M6 approaches were applied to the plasma and CSF LLOQ samples, respectively, and the categorical visual predictive check for censored data showed good agreement between predicted and observed data below the LLOQ. For isoniazid, 118/1,249 (9.4%) of plasma samples and 61/708 (8.6%) of CSF samples were below the LLOQ. The M1 approach was applied to both plasma and CSF LLOQ samples, and the categorical visual predictive check of censored data showed good agreement between predicted and observed data below the LLOQ. For levofloxacin, 10/500 (2.0%) of plasma samples and 6/217 (2.3%) of CSF samples were below LLOQ, and omitted accordingly (M1 approach). For ethambutol and pyrazinamide, 7/584 (1.2%) and 24/1,063 (2.3%) of plasma samples were below the LLOQ, and omitted accordingly (M1 approach).

SUPPLEMENTARY REFERENCES

(1) Van Toi, P. *et al.* High-performance liquid chromatography with time-programmed fluorescence detection for the quantification of Levofloxacin in human plasma and cerebrospinal fluid in adults with tuberculous meningitis. *Journal of chromatography B, Analytical technologies in the biomedical and life sciences* **1061-1062**, 256-62 (2017).

(2) Xu, X.S. *et al*. Impact of low percentage of data below the quantification limit on parameter estimates of pharmacokinetic models. *Journal of pharmacokinetics and pharmacodynamics* **38**, 423-32 (2011).

(3) Dosne, A.G. *et al*. Improving the estimation of parameter uncertainty distributions in nonlinear mixed effects models using sampling importance resampling. *Journal of pharmacokinetics and pharmacodynamics* **43**, 583-96 (2016).

Table S1. Characteristics of the study population at enrolment

	Standard treatment	Intensified treatment	P value
Number of patients	118	115	
HIV positive (%)	50 (42.4)	50 (43.5)	0.970
CD4 (/µL)	40 (2-456)	34.0 (1.0-316)	0.856
Female (%)	38 (32.2)	35 (30.4)	0.881
Age (years)	34.5 (18.0-86.0)	33.0 (18.0-70.0)	0.281
Height (cm)	1.6 (1.5-1.8)	1.6 (1.4-1.8)	0.967
Weight (kg)	50.0 (28.0-73.0)	48.5 (31.0-77.0)	0.511
Glasgow coma scale	14.0 (4.0-15.0)	14.0 (3.0-15.0)	0.879
White cell count (×10^9/L)	8.8 (1.6-22.4)	9.6 (1.5-23.0)	0.670
Hemoglobin (g/L)	12.6 (6.6-16.1)	12.4 (6.8-16.7)	0.888
Platelet (×10^9/L)	291.5 (59.0-556.0)	283.0 (46.0-613.0)	0.468
Creatinine (µmol/L)	57.5 (26.0-118.0)	59.0 (24.0-119.0)	0.929
Bilirubin (µmol/L)	8.0 (2.6-32.7)	8.9 (2.2-36.4)	0.024
Blood glucose (mmol/L)	6.2 (2.7-13.1)	5.8 (3.4-12.3)	0.055
Protein (g/L)	70.2 (0-99.4)	68.9 (0-87.9)	0.085
Albumin (g/L)	37.3 (19.5-49.1)	37.4 (20.6-49.3)	0.765
CSF protein (g/L)	1.5 (0-8.7)	1.7 (0.4-8.7)	0.842
CSF glucose (mmol/L)	1.6 (0.1-7.2)	1.6 (0.2-10.2)	0.624
CSF lactate (mmol/L)	5.6 (1.8-12.0)	5.8 (1.8-13.5)	0.852
CSF white cell count (×10^9/L)	241 (1-2,260)	256 (2-2,302)	0.471
AST (U/L)	28.5 (11.0-153.0)	31.0 (8.0-337.0)	0.616
ALT (U/L)	34.0 (9.0-199.0)	30.0 (7.0-229.0)	0.326
MRC grade (%)			0.994
1	40 (33.9)	39 (33.9)	
2	54 (45.8)	52 (45.2)	
3	24 (20.3)	24 (20.9)	
Resistance category			
Susceptibility results available	73	72	
Isoniazid	18 (24.7)	16 (22.2)	0.120
MDR or rifampin	1 (1.4)	1 (1.4)	ND
No resistance	54 (74.0)	55 (76.4)	0.113
Bacterial load (log) in CSF	3.1 (0.7-5.5)	3.1 (1.9-5.3)	0.613

Continuous variables are reported as median (range), and categorical variables are reported as number (%). Bacterial load are reported in 70 patients each for standard and intensified treatment groups, respectively. CD4 measures are only reported in HIV co-infected patients. P values were calculated using the Kruskal-Wallis test. ND: the test was not done due to very small number of event.

Table S2. Final parameter estimates of levofloxacin population pharmacokinetics in patients with tuberculosis meningitis

Parameter	NONMEM estimates (%RSE)	SIR median (95%CI)	CV for IIV (%RSE)	SIR median (95%CI)	Shrinkage (%)
F (%)	100 *fix*	-	34.6 (8.7)	34.9 (28.9-41.7)	14.0
MTT (h)	0.826 (14.5)	0.820 (0.631-1.095)	82.1 (24.0)	83.2 (62.4-121.2)	44.2
The number of transit comp.	2 *fix*	-	-	-	-
CL/F (L/h)	12.8 (4.6)	12.7 (11.8-13.8)	-	-	
V/F (L)	153.0 (13.6)	153.4 (123.9-190.8)	63.0 (10.9)	62.4 (50.1-77.6)	27.4
Q/F (L/h)	0.0132 (31.7)	0.0133 (0.0075-0.0197)	56.2 (54.7)	58.3 (19.8-102.6)	75.9
V_{csf} (L)	0.15 *fix*	-	-	-	-
PC	0.566 (3.7)	0.565 (0.526-0.604)	-	-	
RUV for plasma	0.131 (5.7)	0.132 (0.112-0.151)	-	-	15.8
RUV for CSF	0.146 (12.0)	0.147 (0.118-0.185)	-	-	13.6
Covariate relationship					
CrCl on CL (%)	0.58 (15.7)	0.58 (0.41-0.75)	-		-
Secondary parameters					
Day 14 $C_{max\ plasma}$ (mg/L)	8.9 (2.5-16.7)				
Day 14 AUC_{plasma} (h×mg/L)	70.8 (24.1-137.2)				
Day 14 $C_{max\ CSF}$ (mg/L)	3.6 (1.2-7.0)				
Day 14 AUC_{CSF} (h×mg/L)	40.1 (13.6-77.7)				

F is the relative bioavailability. CL/F is the elimination clearance. V/F is the central volume of distribution. MTT is the mean transit time. Q/F is the inter-compartmental clearance. V_{CSF}/F is the CSF volume of distribution. PC is the partition coefficient between central and CSF compartment. CrCl is the creatinine clearance. CrCl was included on parameter CL using a linear model ($CL = CL_{TV} \cdot (1 + \theta \cdot (CrCl - 106))$), and CL_{TV} is the typical CL value of the population. RUV is the additive residual error on log scale. C_{max} is the peak concentration. AUC is the area under the concentration-time curve. SIR is the sampling importance resampling. Population estimates in the table are given for a “typical” patient with free fat mass of 70 kg. The calculation of IIV (interindividual variability) and RSE (relative standard error), as well as secondary parameters refer to Table 1.

Table S3. Final parameter estimates of ethambutol population pharmacokinetics in patients with tuberculosis meningitis

Parameter	NONMEM estimates (%RSE)	SIR median (95%CI)	CV for IIV (%RSE)	SIR median (95%CI)	Shrinkage (%)
F (%)	100 *fix*	-	-	-	-
MTT (h)	1.55 (10.1)	1.52 (1.28-1.94)	61.1 (12.7)	61.8 (47.2-76.9)	35.5
The number of transit comp.	1 *fix*	-	-	-	-
CL/F (L/h)	80.3 (2.9)	80.4 (76.1-84.7)	19.1 (27.2)	19.4 (13.1-25.8)	40.7
V2/F (L)	278 (41.6)	285 (201-349)	48.5 (26.6)	49.6 (28.0-69.4)	60.7
Q/F (L/h)	88.9 (12.3)	88.0 (74.1-106.8)	-	-	-
V3/F (L)	707 (10.2)	704 (598-882)	-	-	-
RUV	0.153 (11.3)	0.154 (0.134-0.179)	-	-	11.3
Secondary parameters					
Day 14 C_{max} (mg/L)	2.4 (1.1-5.4)				
Day 14 AUC (h×mg/L)	15.8 (10.7-25.6)				

F is the relative bioavailability. CL/F is the elimination clearance. V2/F is the central volume of distribution. MTT is the mean transit time. Q/F is the inter-compartmental clearance. V3/F is the peripheral volume of distribution. RUV is the additive residual error on log scale. SIR is the sampling importance resampling. Population estimates in the table are given for a "typical" patient with free fat mass of 70 kg. C_{max} is the peak concentration. AUC is the area under the concentration-time curve. The calculation of IIV and RSE, as well as secondary parameters refer to Table 1.

Table S4. Final parameter estimates of pyrazinamide population pharmacokinetics in patients with tuberculosis meningitis

**Parameter**	**NONMEM estimates (%RSE)**	**SIR median (95%CI)**	**CV for IIV (%RSE)**	**SIR median (95%CI)**	**Shrinkage (%)**
F (%)	100 *fix*	-	-	-	-
MTT (h)	0.240 (28.1)	0.241 (0.170-0.311)	102.5 (15.7)	102.2 (78.6-132.1)	60.6
The number of transit comp.	2 *fix*	-	-	-	-
CL/F (L/h)	4.88 (4.5)	4.88 (4.57-5.21)	42.2 (17.7)	42.5 (37.9-47.6)	13.6
V/F (L)	62.2 (9.7)	62.4 (56.3-67.7)	68.6 (19.4)	68.6 (60.5-77.6)	16.6
RUV	0.0312 (13.7)	0.0313 (0.0277-0.0352)	-	-	21.2
Covariate relationship					
AST on CL (%)	-0.36 (27.6)	-0.35 (-0.45 to -0.25)	-	-	-
Secondary parameters					
Day 14 C_{max} (mg/L)	40.7 (4.9-106.9)				
Day 14 AUC (h×mg/L)	378.6 (73.3-1,349.2)				

F is the relative bioavailability. CL/F is the elimination clearance. V/F is the central volume of distribution. MTT is the mean transit time. AST is the aspartate aminotransferase. AST was included on parameter CL using an exponential model($CL = CL_{TV} \cdot e^{\theta \cdot (AST-30)}$, and CL_{TV} is the typical CL value of the population). RUV is the additive residual error on log scale. SIR is the sampling importance resampling. Population estimates in the table are given for a "typical" patient with free fat mass of 70 kg. C_{max} is the peak concentration. AUC is the area under the concentration-time curve. The calculation of IIV and RSE, as well as secondary parameters refer to Table 1.

Table S5. Baseline characteristics by outcome

	Survival	Death	P value
Number of patients	195	38	
HIV positive	76 (39.0)	24 (63.2)	0.010
CD4 (/µL)	39 (2-456)	19 (1-158)	0.113
Female	62 (31.8)	11 (28.9)	0.877
Intensified treatment	93 (47.7)	22 (57.9)	0.330
Age (years)	34.0 (18.0-70.0)	35.0 (19.0-86.0)	0.272
Height (cm)	1.6 (1.4-1.8)	1.6 (1.5-1.7)	0.916
Weight (kg)	50.0 (28.0-77.0)	45.0 (35.0-70.0)	0.003
Glasgow coma scale	14.0 (6.0-15.0)	10.5 (3.0-15.0)	<0.001
White cell count (×10^9/L)	9.6 (1.5-23.0)	8.3 (1.6-21.7)	0.237
Hemoglobin (g/L)	12.6 (6.6-16.1)	11.7 (6.8-16.7)	0.121
Platelet (×10^9/L)	290.0 (46.0-613.0)	256.0 (47.0-527.0)	0.061
Creatinine (µmol/L)	59.0 (24.0-119.0)	55.0 (35.0-118.0)	0.564
Bilirubin (µmol/L)	8.1 (2.2-35.3)	10.2 (3.5-36.4)	0.038
Blood glucose (mmol/L)	5.9 (2.7-13.1)	6.6 (3.4-12.9)	0.032
Protein (g/L)	69.7 (0-99.4)	66.0 (0-85.4)	0.015
Albumin (g/L)	38.2 (20.6-49.3)	32.4 (19.5-48.7)	<0.001
CSF protein (g/L)	1.6 (0-8.7)	1.6 (0.3-7.7)	0.964
CSF glucose (mmol/L)	1.6 (0.1-7.2)	1.7 (0.2-10.2)	0.281
CSF lactate (mmol/L)	5.6 (1.8-11.8)	6.0 (1.8-13.5)	0.406
CSF white cell count (×10^9/L)	257.0 (2.0-2302.0)	217.5 (1.0-860.0)	0.321
AST (U/L)	28.0 (11.0-176.0)	42.0 (8.0-337.0)	<0.001
ALT (U/L)	28.0 (7.0-181.0)	43.0 (14.0-229.0)	0.004
MRC grade (%)			<0.001
1	70 (35.9)	9 (23.7)	
2	97 (49.7)	9 (23.7)	
3	28 (14.4)	20 (52.6)	
Isoniazid fast metabolizer	113 (57.9)	28 (71.8)	0.07
Resistance category			
Susceptibility results available	119	26	
Isoniazid	30 (25.2)	4 (15.4)	0.284
MDR or rifampicin	1 (0.8)	1 (3.8)	ND
No resistance	88 (73.9)	21 (80.8)	0.466
Bacterial load (log) in CSF	3.1 (0.7-5.5)	3.3 (1.4-4.8)	0.149

Continuous variables are reported as median (range), and categorical variables are reported as number (%). Bacterial load are reported in 111 and 29 patients for survival and death outcome, respectively. CD4 measures were only reported in HIV co-infected patients. P values were calculated using the Kruskal-Wallis test. ND: the test was not done due to very small number of event.

Table S6. Accuracy and precision of developed assays to quantify the 4 anti- tuberculosis drugs in plasma and CSF.

	Reportable range (ng/mL)	Accuracy and Precision of quality control (QC) samples		
		Low QC	Medium QC	High QC
Plasma samples				
Rifampin	8.00-18,400	99.6 (8.57)	97.4 (4.71)	97.3 (5.95)
Pyrazinamide	800-57,500	98.6 (8.89)	101 (7.37)	99.0 (7.88)
Isoniazid	12.0-13,800	105 (14.6)	103 (9.37)	103 (8.10)
EMB	8.00-4,830	102 (7.78)	101 (4.52)	95.4 (5.64)
CSF samples				
Rifampin	32.0-1,600	104 (9.30)	102 (8.16)	103 (6.77)
Isoniazid	36.0-4,600	107 5.26)	110 (4.75)	106 (4.90)

QC data are presented as accuracy% (RSD%).

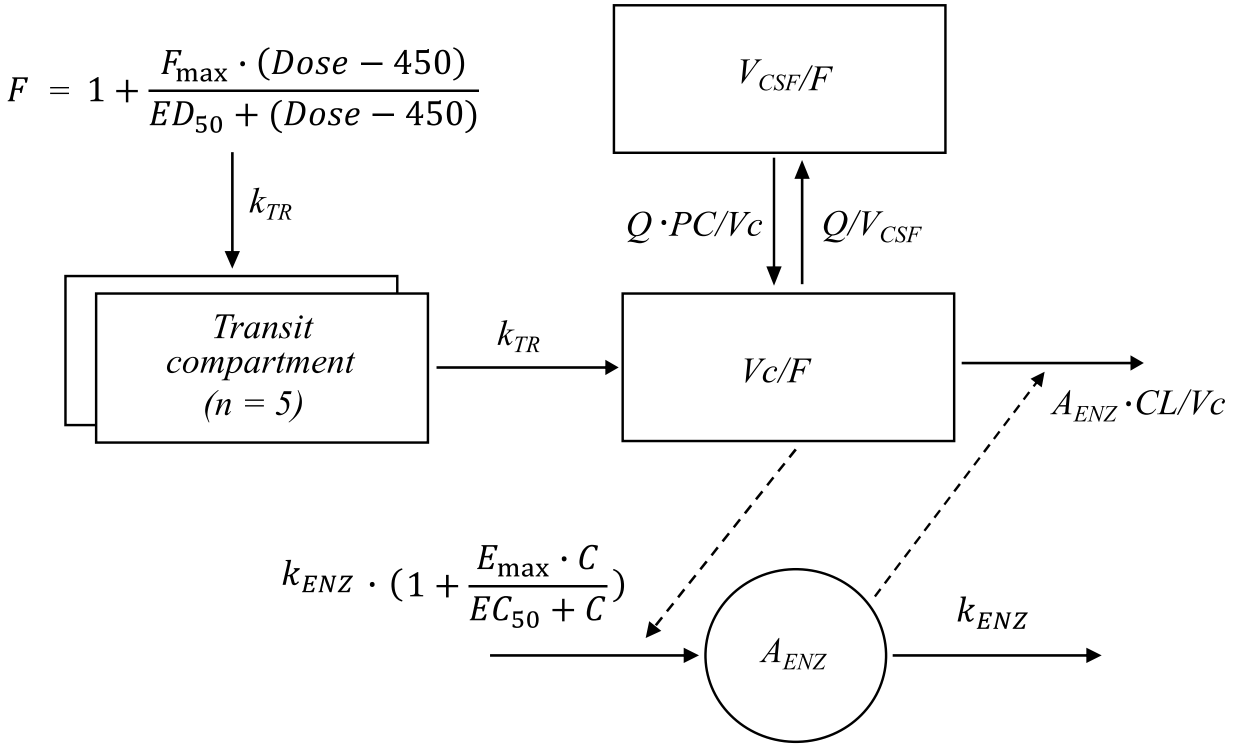

Figure S1. Graphical overview of the structural pharmacokinetic model for rifampin.

CSF is the cerebrospinal fluid. F is the relative bioavailability. CL is the elimination clearance. Vc is the plasma volume of distribution. MTT is the mean transit time. E_{max} is the maximum increase in enzyme formation rate. k_{enz} is the enzyme degradation rate. EC_{50} is the plasma concentration corresponding to 50% of E_{max}. F_{max} is the maximum increase in relative bioavailability and ED_{50} is the dose corresponding to half of the F_{max}. Q is the inter-compartmental clearance. V_{CSF} is the CSF volume of distribution. PC is the partition coefficient between central and CSF compartment.

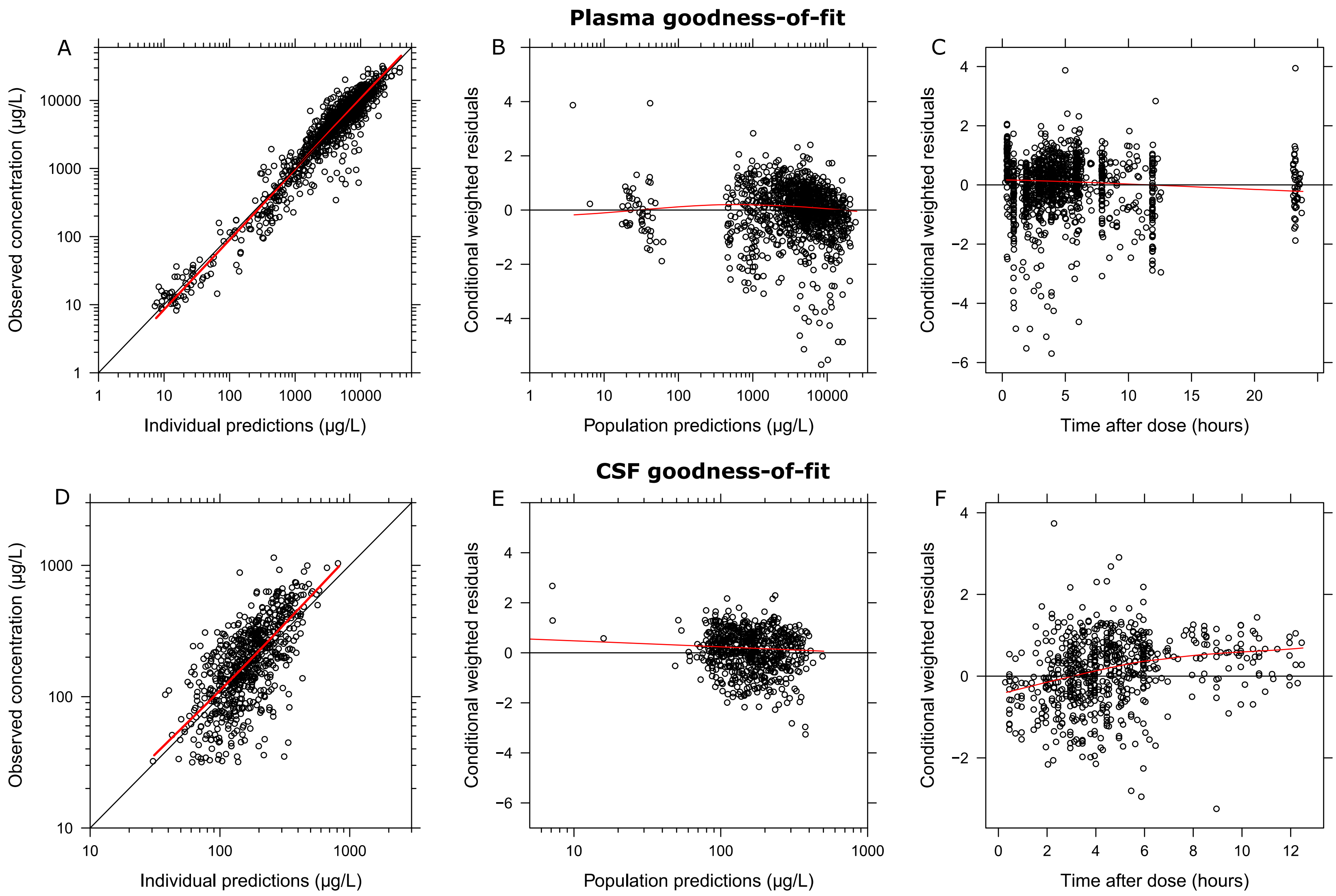

Figure S2. Goodness-of-fit of the final population pharmacokinetic model describing rifampin in plasma (A, B, C) and CSF (D, E, F).

Observed plasma concentrations vs. individually predicted concentrations (A, D). Conditionally weighted residuals vs. population predicted concentrations (B, E). Conditionally weighted residuals vs. time (C, F). Solid red lines represent locally weighted least squares regressions.

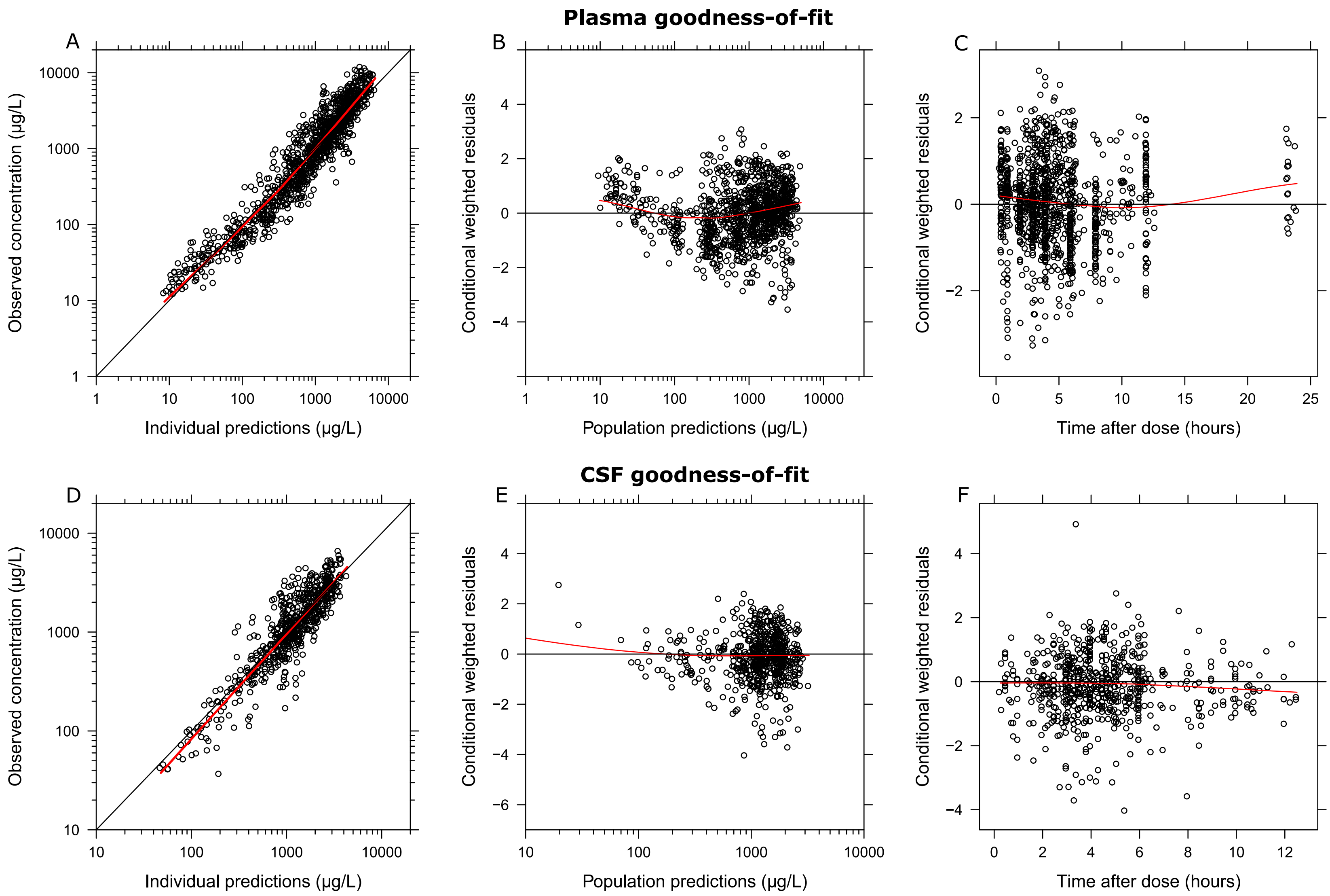

Figure S3. Goodness-of-fit of the final population pharmacokinetic model describing isoniazid in plasma (A, B, C) and CSF (D, E, F).

Observed plasma concentrations vs. individually predicted concentrations (A, D). Conditionally weighted residuals vs. population predicted concentrations (B, E). Conditionally weighted residuals vs. time (C, F). Solid red lines represent locally weighted least squares regression.

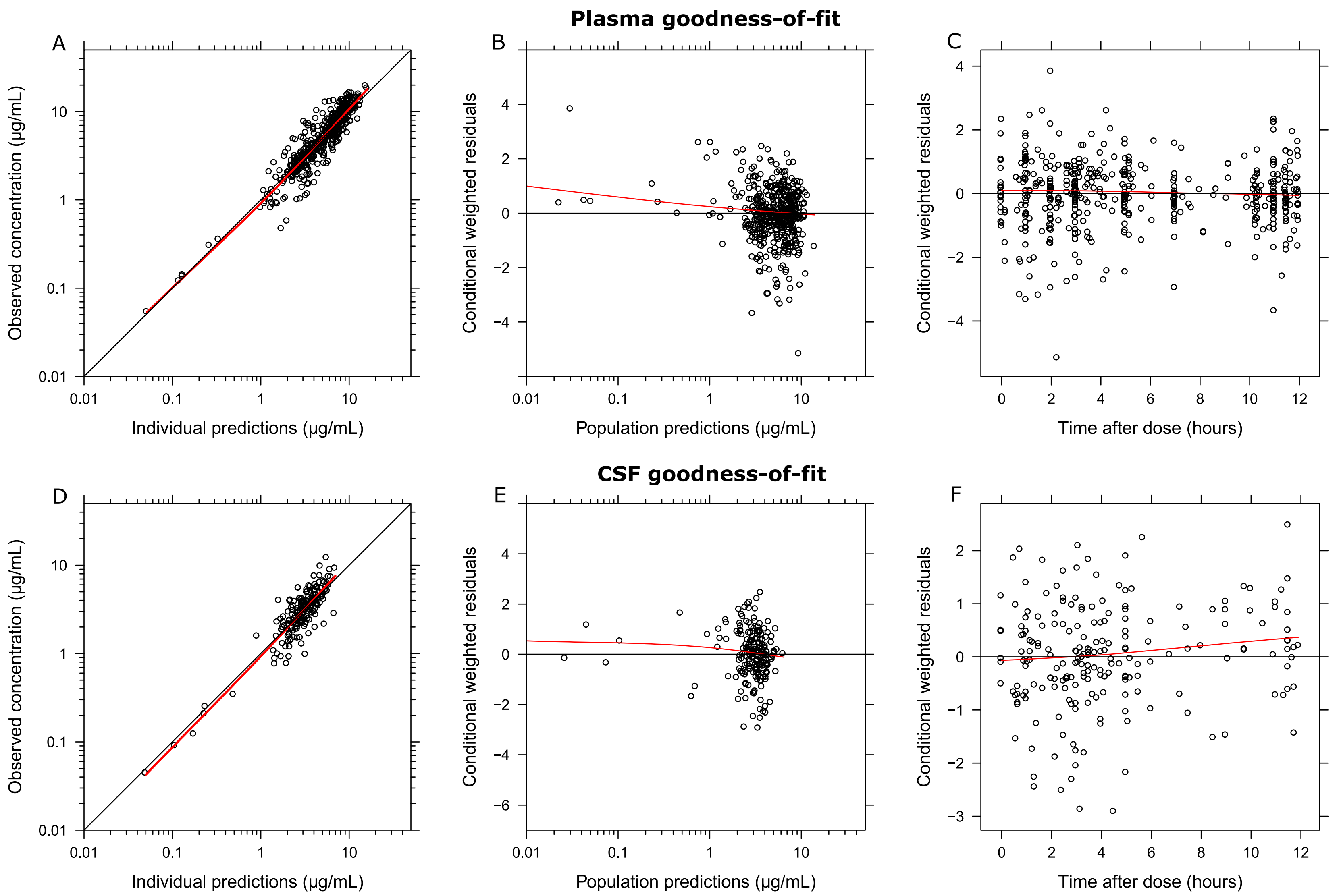

Figure S4. Goodness-of-fit of the final population pharmacokinetic model describing levofloxacin in plasma (A, B, C) and CSF (D, E, F).

Observed plasma concentrations vs. individually predicted concentrations (A, D). Conditionally weighted residuals vs. population predicted concentrations (B, E). Conditionally weighted residuals vs. time (C, F). Solid red lines represent locally weighted least squares regression.

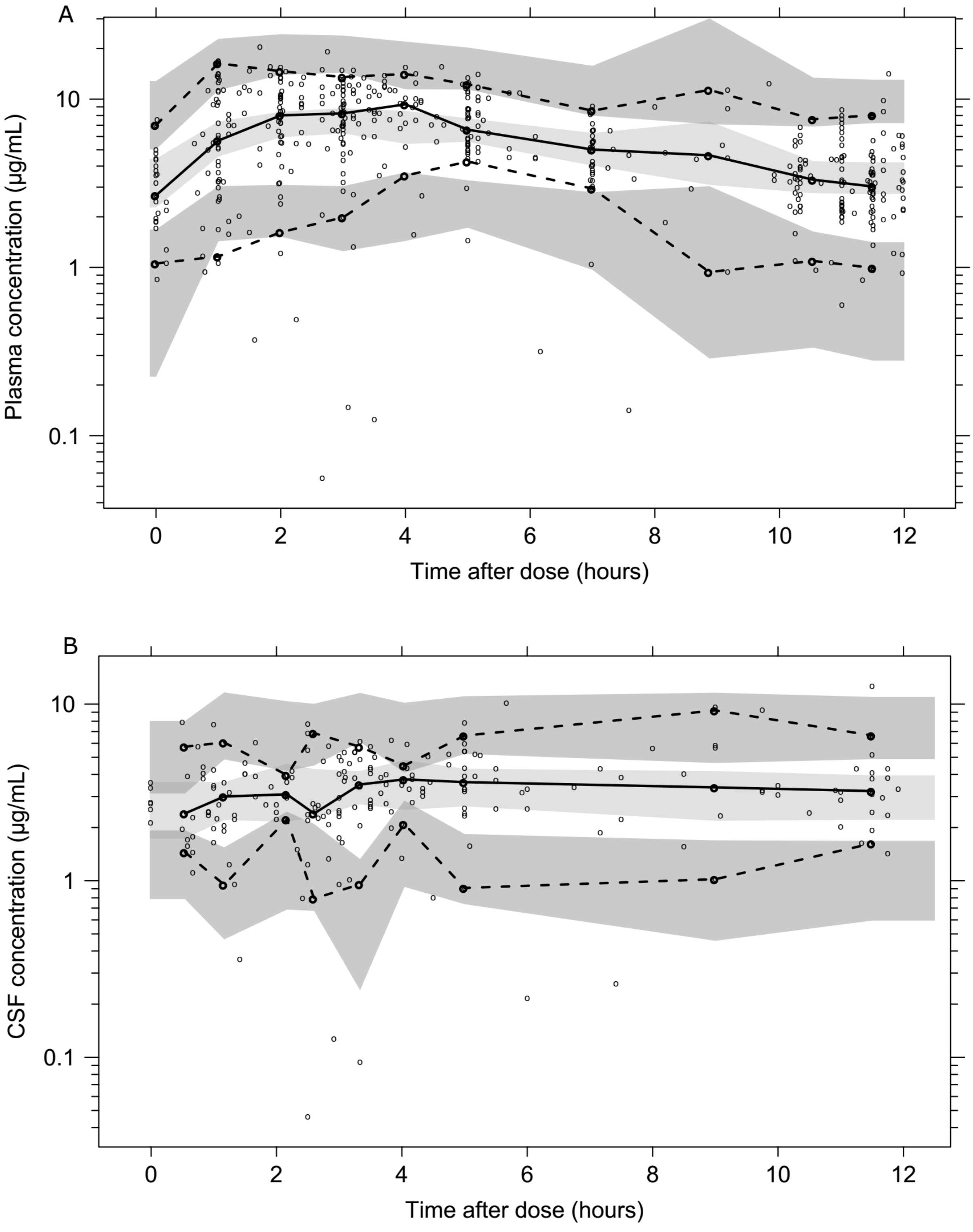

Figure S5. Visual predictive check of the final population pharmacokinetic model for levofloxacin in plasma (A) and CSF (B) based on 1,000 stochastic simulations.

Open circles represent the observations. Solid lines represent the 5th, 50th, and 95th percentiles of the observed data. The shaded areas represent the 95% confidence intervals around the simulated 5th, 50th, and 95th percentiles.

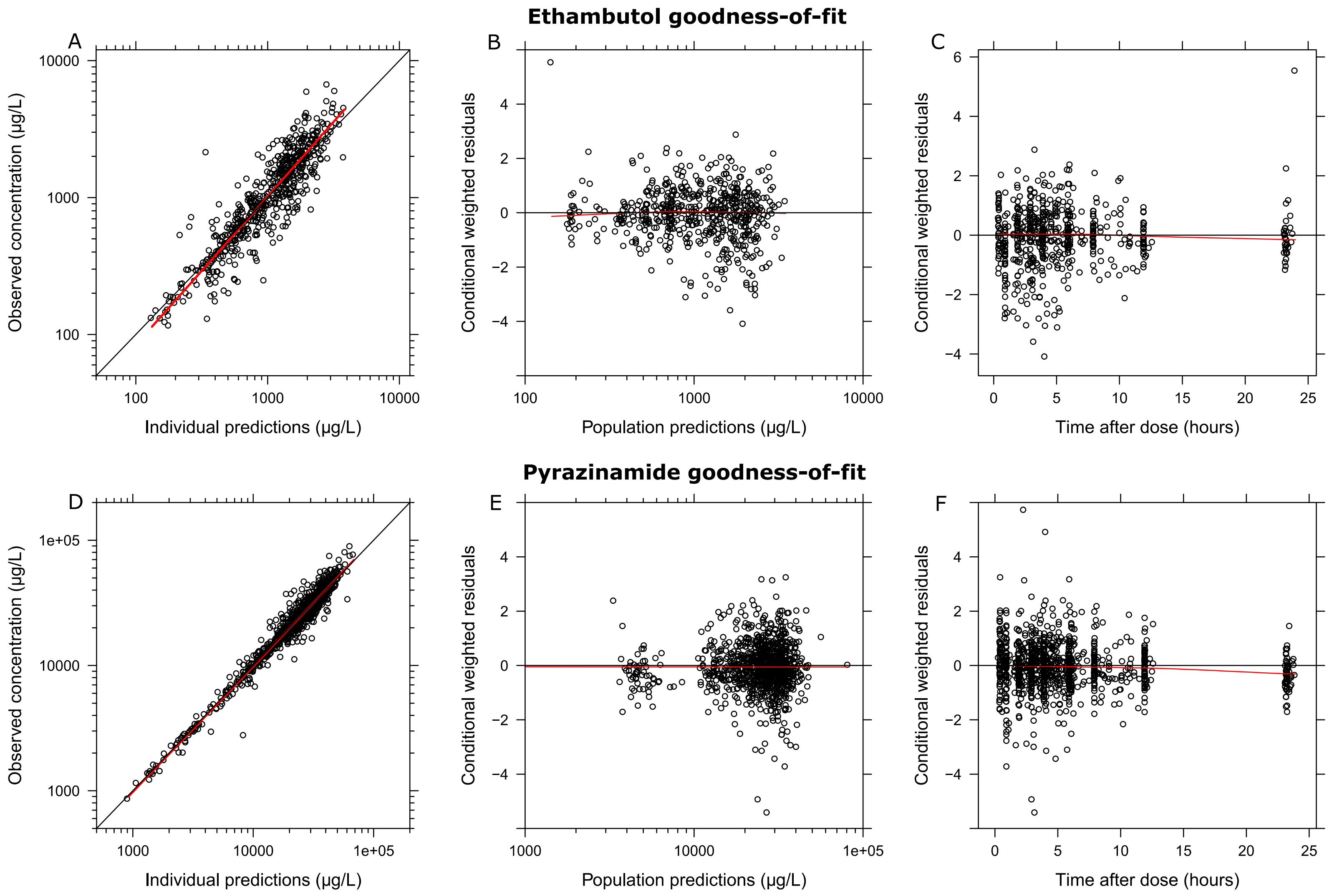

Figure S6. Goodness-of-fit of the final population pharmacokinetic model describing ethambutol (A, B, C) and pyrazinamide (D, E, F) in plasma.

Observed plasma concentrations vs. individually predicted concentrations (A, D). Conditionally weighted residuals vs. population predicted concentrations (B, E). Conditionally weighted residuals vs. time (C, F). Solid red lines represent locally weighted least squares regression.

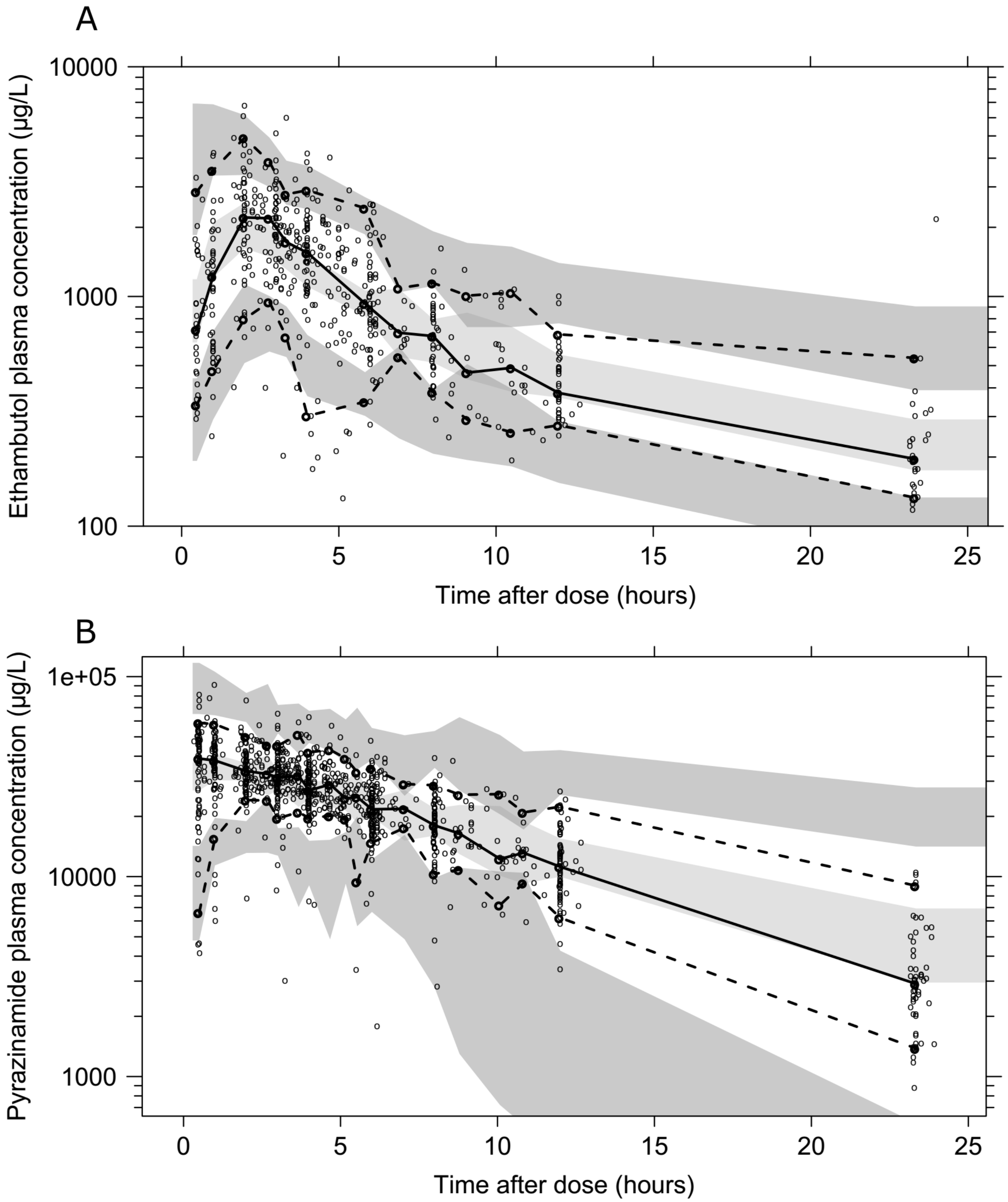

Figure S7. Visual predictive check of the final population pharmacokinetic model for ethambutol (A) and pyrazinamide (B) in plasma based on 1,000 stochastic simulations.

Open circles represent the observations. Solid lines represent the 5th, 50th, and 95th percentiles of the observed data. The shaded areas represent the 95% confidence intervals around the simulated 5th, 50th, and 95th percentiles.

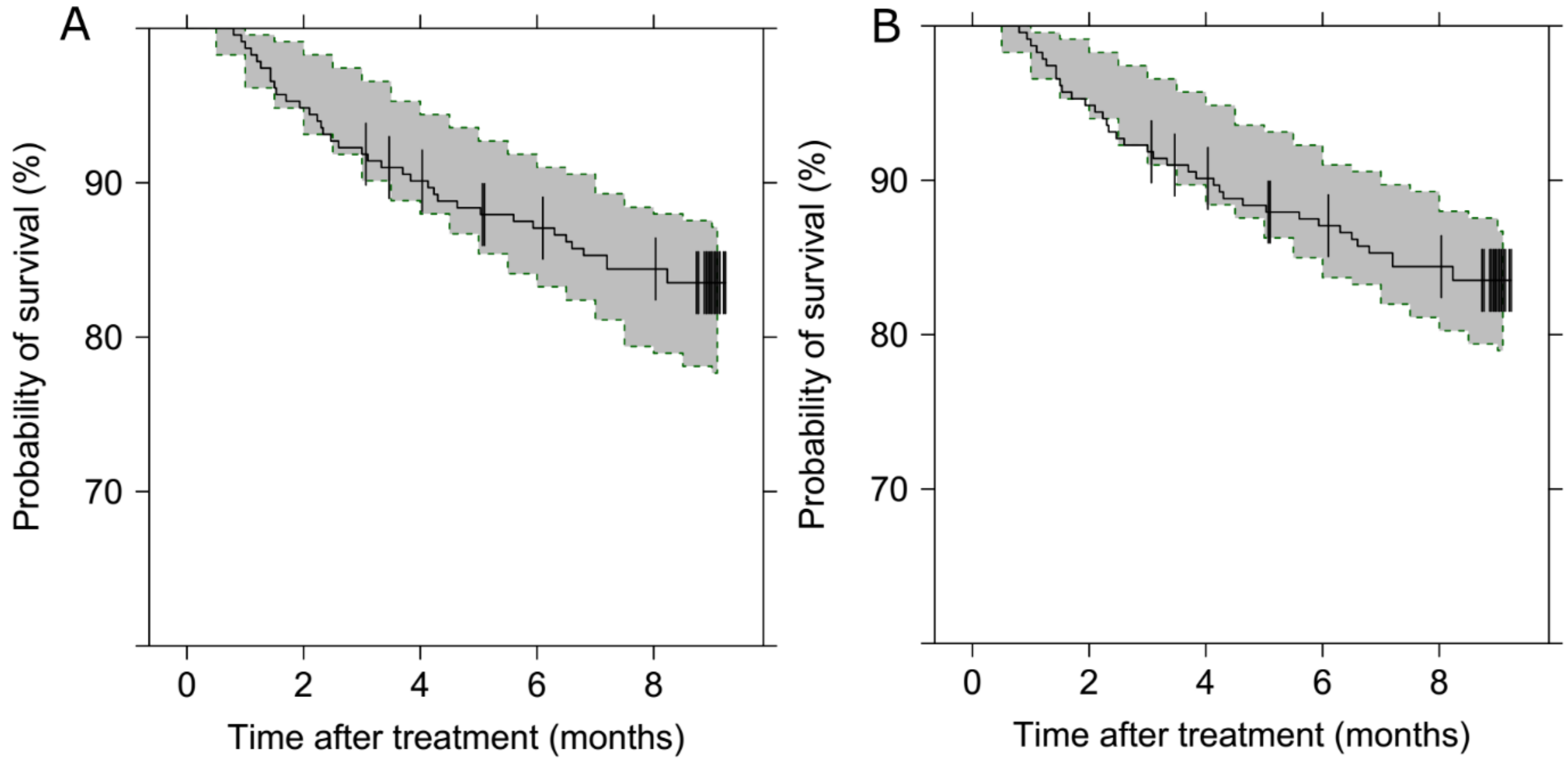

Figure S8. Visual predictive check of the time-to-event model describing time to death using either isoniazid C_{max} (A) or $AUC_{0\text{-}24}$ (B) at steady state (day 14) as a descriptor of hazard.

Black solid lines represent the observed Kaplan-Meier plots. Shaded areas represent the simulated 95% prediction interval from the final pharmacodynamic model.

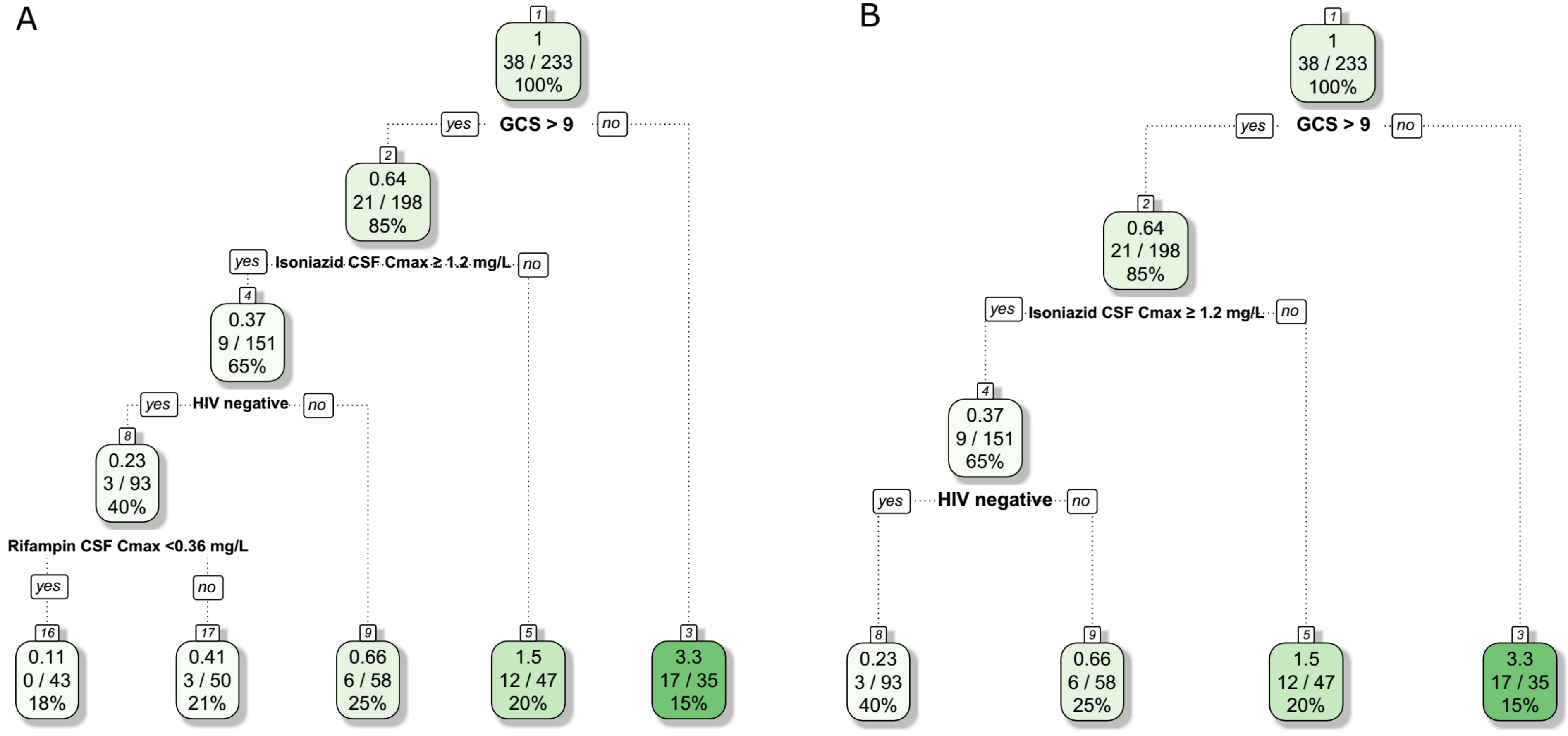

Figure S9. The classification and regression tree model for predicting death in patients.

The full tree and the final tree are shown in A and B, respectively. The average predicted response (yval) is shown in each node. The response at the root node was assumed to be 1. A higher predicted response is associated with a higher risk of death. The numerators indicate number of deaths, and the denominators represent the total number of patients in each node. The percentage in each node is the fraction of patients in the node vs. total patients.
