# Supplementary material for: Pharmacokinetics and Pharmacodynamics of Intensive Antituberculosis Treatment of Tuberculous Meningitis
Source: Clin Pharmacol Ther. 2020 Feb 29;107(4):1023–33. doi: 10.1002/cpt.1783 (PMC7158205; doi:10.1002/cpt.1783)
Supplement: Supplementary file 2 — NONMEM Code. [file CPT-107-1023-s002.pdf]

NONMEM code for final rifampin population PK model

```
$INPUT
        ID              ; Patient ID
        TIME            ; Time of sample/dose
        DV              ; Dependent variable (natural logarithm of observed concentrations, µg/L)
        WT              ; Body weight (kg, covariate)
        SEX             ; Age (months, covariate)
        DOSE            ; Dose (mg, covariate)
        CSFP            ; CSF protein (g/L, covariate)
        EVID            ; Event ID record
        MDV             ; Missing dependent variable (1=missing)
        AMT             ; Dose amount (mg)
        CMT             ; Compartment

$DATA
        dataset.csv  IGNORE=#

$SUBROUTINE
        ADVAN6 TOL=9

$MODEL
        COMP = (1)              ; Dose compartment
        COMP = (2)              ; Central compartment
        COMP = (3)              ; CSF compartment
        COMP = (4)              ; Enzyme compartment
        COMP = (5)              ; Transit compartment 1
        COMP = (6)              ; Transit compartment 2
        COMP = (7)              ; Transit compartment 3
        COMP = (8)              ; Transit compartment 4
        COMP = (9)              ; Transit compartment 5
```

```
$PK
;------------------------------------ Dose covariate ----------------------------------------------------------------------
        FEMAX = THETA(11)                                       ; Maximum dose effect on relative bioavailability
        FD50 = THETA(12)                                        ; Dose to reach 50% of maximum effect
        CDOSE = 1 + FEMAX * (DOSE-450)/(FD50+(DOSE-450))        ; Dose covariate relationship
;-------------------------------------------------------------------------------------------------------------------------------

;------------------------------------ CSF protein covariate----------------------------------------------------------------
        CCSFP = 1 + (CSFP - 1.6) * THETA(13)                    ; Linear covariate relationship for CSF protein
;-------------------------------------------------------------------------------------------------------------------------------

;------------------------------------ Lean bodyweight-------------------------------------------------------------------------
        BMI = WT/(HT**2)                                        ; Calculation of BMI
        IF (SEX.EQ.1) THEN
          LBW = (9270*WT)/(6680+216*BMI)                        ; Lean bodyweight for males
        ENDIF
        IF (SEX.EQ.2) THEN
          LBW = (9270*WT)/(8780+244*BMI)                        ; Lean bodyweight for females
        ENDIF
;-------------------------------------------------------------------------------------------------------------------------------
        TVCL = THETA(1) * ((LBW/70)**0.75)                      ; Population clearance
        CL = TVCL * EXP(ETA(1))                                 ; Individual clearance

        TVV2 = THETA(2) * (LBW/70)                              ; Population central volume
        V2 = TVV2 * EXP(ETA(2))                                 ; Individual central volume

        TVMT = THETA(3)                                         ; Population mean transit time
        MT = TVMT * EXP(ETA(3))                                 ; Individual mean transit time

        TVF1 = THETA(4) * CDOSE                                 ; Population relative bioavailability
        F1 = TVF1 * EXP(ETA(4))                                 ; Individual relative bioavailability
```

```
TVEMAX  = THETA(5)                      ; Maximum increase in enzyme formation rate
EMAX = TVEMAX * EXP(ETA(5))             ; Individual Emax

TVEC50  = THETA(6)                      ; Plasma concentration to reach 50% of Emax
EC50  = TVEC50  * EXP(ETA(6))           ; Individual EC50

TVKENZ  = THETA(7)                      ; Population enzyme degradation rate
KENZ  = TVKENZ * EXP(ETA(7))            ; Individual enzyme degradation rate

TVQ = THETA(8)                          ; Population inter-compartment clearance
Q = TVQ * EXP(ETA(8))                   ; Individual inter-compartment clearance

TVPC  = THETA(9)*CCSFP                  ; Population partition coefficient
PC = TVPC * EXP(ETA(9))                 ; Individual partition coefficient

TVV3 = THETA(10) * (LBW/70)             ; Population CSF volume
V3 = TVV3 * EXP(ETA(10))                ; Individual CSF volume

NN= 5                                   ; Number of transit compartments
KTR = (NN + 1) / MT                     ; Transit rate constant
K15 = KTR                               ; Transit rate constant (COMP 1 --> 5)
K56 = KTR                               ; Transit rate constant (COMP 5 --> 6)
K67 = KTR                               ; Transit rate constant (COMP 6 --> 7)
K78 = KTR                               ; Transit rate constant (COMP 7 --> 8)
K89 = KTR                               ; Transit rate constant (COMP 8 --> 9)
K92 = KTR                               ; Transit rate constant (COMP 9 --> 2)
K20 = CL/V2                             ; Elimination rate constant (COMP 2 --> 0)
K23 = Q * PC/V2                         ; Distribution rate constant (COMP 2 --> 3)
K32 = Q/V3                              ; Distribution rate constant (COMP 3 --> 2)
S2 = V2/1000                            ; Scaling for central volume
S3 = V3/1000                            ; Scaling for CSF volume
```

```
$DES
	CP = A(2)/V2                                          ; Predicted plasma concentration
	EFF = (EMAX * CP) / (EC50 + CP)                       ; Effect of plasma concentration
	DADT (1) = -A(1) * K15                                ; 1 Dose compartment
	DADT (2) = A(9) * K92 - A(2) * A(4) * K20 - A(2)*K23 + A(3)*K32     ; 2 Central compartment
	DADT (3) = A(2)*K23 - A(3)*K32                        ; 3 CSF compartment
	DADT (4) = KENZ * (1 + EFF) - KENZ * A(4)             ; 4 Enzyme compartment
	DADT (5) = A(1) * K15 - A(5) * K56                    ; 5 Transit compartment 1
	DADT (6) = A(5) * K56 - A(6) * K67                    ; 6 Transit compartment 2
	DADT (7) = A(6) * K67 - A(7) * K78                    ; 7 Transit compartment 3
	DADT (8) = A(7) * K78 - A(8) * K89                    ; 8 Transit compartment 4
	DADT (9) = A(8) * K89 - A(9) * K92                    ; 9 Transit compartment 5

$ERROR
	IF(F.GT.0)  IPRED = LOG(F)                            ; Natural logarithm of predictions
	IF (CMT.EQ.2)    W = SQRT(SIGMA(1,1))                 ; Plasma concentration residual error
	IF (CMT.EQ.3)    W = SQRT(SIGMA(2,2))                 ; CSF concentration residual error
	IRES = IPRED – DV                                     ; Individual residual error
	IWRES = IRES / W                                      ; Individual weighted residual error
	IF (CMT.EQ.2)    Y = IPRED + EPS(1)                   ; Plasma concentration additive residual error
	IF (CMT.EQ.3)    Y = IPRED + EPS(2)                   ; CSF concentration additive residual error

$THETA                                                        ; Initial estimates of theta
	(0, 10.1)                                             ; 1. Clearance
	(0, 76)                                               ; 2. Central volume of distribution
	(0, 0.99)                                             ; 3. Mean transit time
	(1) FIX                                               ; 4. Relative bioavailability
	(1.16) FIX                                            ; 5. Maximum increase in enzyme formation rate
	(0.0699) FIX                                          ; 6. Plasma concentration to reach 50% of Emax
	(0.00603) FIX                                         ; 7. Enzyme degradation rate
	(0, 0.00387)                                          ; 8. Inter-compartment clearance
	(0, 0.0712)                                           ; 9. Partition coefficient
	(0.15) FIX                                            ; 10. CSF volume of distribution
```

```
        (0.504) FIX                          ; 11. Maximum effect on relative bioavailability
        (67) FIX                             ; 12. Dose to reach 50% of maximum effect
        (0, 0.141)                           ; 13. CSF protein effect on PC

$OMEGA                                       ; Initial estimates for omega
        0.115                                ; 1. Clearance
        0.0462                               ; 2. Central volume of distribution
        0.474                                ; 3. Mean transit time
        0.11                                 ; 4. Relative bioavailability
        0 FIX                                ; 5. Maximum increase in enzyme formation rate
        0 FIX                                ; 6. Plasma concentration to reach 50% of Emax
        0 FIX                                ; 7. Enzyme degradation rate
        0.924                                ; 8. Inter-compartment clearance
        0 FIX                                ; 9. Partition coefficient
        0 FIX                                ; 10 CSF volume of distribution

$SIGMA                                       ; Initial estimates of sigma
        (0.253)                              ; 1. Plasma concentration residual variability
        (0.49)                               ; 2. CSF concentration residual variability

$ESTIMATION   POSTHOC MAXEVAL=9999   METHOD=1 INTER
```

NONMEM code for final isoniazid population PK model

```
$INPUT
        ID              ; Patient ID
        TIME            ; Time of sample/dose
        DV              ; Dependent variable (natural logarithm of observed concentrations, µg/L)
        WT              ; Body weight (kg, covariate)
        SEX             ; Age (months, covariate)
        EVID            ; Event ID record
        MDV             ; Missing dependent variable (1=missing)
        AMT             ; Dose amount (mg)
        CMT             ; Compartment

$DATA
        dataset.csv  IGNORE=#

$ABBREVIATED COMRES=4

$SUBROUTINE
        ADVAN6 TOL=9

$MODEL
        COMP = (1)              ; Dose compartment
        COMP = (2)              ; Central compartment
        COMP = (3)              ; CSF compartment
        COMP = (4)              ; Transit compartment 1
        COMP = (5)              ; Transit compartment 2
        COMP = (6)              ; Transit compartment 3
        COMP = (7)              ; Transit compartment 4
        COMP = (8)              ; Transit compartment 5
        COMP = (9)              ; Plasma AUC compartment
        COMP = (10)             ; CSF AUC compartment
```

```
$MIX
        NSPOP = 2                   ; Number of sub-population
        PMIX   = THETA(9)           ; Probability
        P(2)  = PMIX                ; Probability of subpopulation 2
        P(1)  = 1-PMIX              ; Probability of subpopulation 1
$PK
;----------------------------------- Lean bodyweight---------------------------------------------------------------------------
        BMI = WT/(HT**2)                                ; Calculation of BMI
        IF (SEX.EQ.1) THEN
          LBW = (9270*WT)/(6680+216*BMI)                ; Lean bodyweight for males
        ENDIF
        IF (SEX.EQ.2) THEN
          LBW = (9270*WT)/(8780+244*BMI)                ; Lean bodyweight for females
        ENDIF
;----------------------------------------------------------------------------------------------------------------------------- -------------
        IF (MIXNUM.EQ.1)  THEN
          TVCL = THETA (1) * (LBW/70) ** 0.75           ; Clearance for subpopulation 1
        ENDIF
        IF (MIXNUM.EQ.2)  THEN
          TVCL = THETA (2) * (LBW/70) ** 0.75           ; Clearance for subpopulation 2
        ENDIF
        CL = TVCL * EXP(ETA(1))                         ; Individual clearance

        TVV2 = THETA(3) * (LBW/70)                      ; Population central volume
        V2 = TVV2 * EXP(ETA(2))                         ; Individual central volume

        TVMT = THETA(4)                                 ; Population mean transit time
        MT = TVMT * EXP(ETA(3))                         ; Individual mean transit time

        TVF1 = THETA(5)                                 ; Population relative bioavailability
        F1 = TVF1 * EXP(ETA(4))                         ; Individual relative bioavailability
```

```
    TVQ = THETA(6)                          ; Population inter-compartment clearance
    Q = TVQ * EXP(ETA(5))                   ; Individual inter-compartment clearance

    TVPC  = THETA(7)                        ; Population partition coefficient
    PC = TVPC * EXP(ETA(6))                 ; Individual partition coefficient

    TVV3 = THETA(8) * (LBW/70)              ; Population CSF volume
    V3 = TVV3 * EXP(ETA(7))                 ; Individual CSF volume

    NN= 5                                   ; Number of transit compartments
    KTR = (NN + 1) / MT                     ; Transit rate constant
    K14 = KTR                               ; Transit rate constant (COMP 1 --> 4)
    K45 = KTR                               ; Transit rate constant (COMP 4 --> 5)
    K56 = KTR                               ; Transit rate constant (COMP 5 --> 6)
    K67 = KTR                               ; Transit rate constant (COMP 6 --> 7)
    K78 = KTR                               ; Transit rate constant (COMP 7 --> 8)
    K82 = KTR                               ; Transit rate constant (COMP 8 --> 2)
    K20 = CL/V2                             ; Elimination rate constant (COMP 2 --> 0)
    K23 = Q * PC/V2                         ; Distribution rate constant (COMP 2 --> 3)
    K32 = Q/V3                              ; Distribution rate constant (COMP 3 --> 2)
    S2 = V2/1000                            ; Scaling for central volume
    S3 = V3/1000                            ; Scaling for CSF volume

    IF(NEWIND.LE.1) THEN                    ; Assign negative value for the new subject
      COM(1)=-1                             ; Holder of plasma Cmax
      COM(2)=-1                             ; Holder of plasma Tmax
      COM(3)=-1                             ; Holder of CSF Cmax
      COM(4)=-1                             ; Holder of CSF Tmax
    ENDIF

$DES
    DADT (1) = -A(1) * K14                  ; 1 Dose compartment
    DADT (2) = A(8) * K82 - A(2) * K20 - A(2)*K23 + A(3)*K32  ; 2 Central compartment
```

```
    DADT (3) = A(2)*K23 - A(3)*K32                 ; 3 CSF compartment
    DADT (4) = A(1) * K14 - A(4) * K45             ; 4 Transit compartment 1
    DADT (5) = A(4) * K45 - A(5) * K56             ; 5 Transit compartment 2
    DADT (6) = A(5) * K56 - A(6) * K67             ; 6 Transit compartment 3
    DADT (7) = A(6) * K67 - A(7) * K78             ; 7 Transit compartment 4
    DADT (8) = A(7) * K78 - A(8) * K82             ; 8 Transit compartment 8

    DADT (9) = A(2)                                ; 9 Accumulated plasma amount
    AUC_P = A(9)/S2                                ; Plasma AUC
    DADT (10) = A(3)                               ; 10 Accumulated CSF amount
    AUC_C = A(10)/S3                               ; CSF AUC

    CT=A(2)/S2                                      ; Plasma concentration
    IF(CT.GT.COM(1)) THEN
      COM(1)=CT                                    ; Plasma Cmax
      COM(2)=T                                     ; Plasma Tmax
    ENDIF

    CCSF=A(3)/S3                                   ; CSF concentration
    IF(CCSF.GT.COM(3)) THEN
      COM(3)=CCSF                                  ; CSF Cmax
      COM(4)=T                                     ; CSF Tmax
    ENDIF

$ERROR
    IF(F.GT.0)  IPRED = LOG(F)                     ; Natural logarithm of predictions
    IF (CMT.EQ.2)    W = SQRT(SIGMA(1,1))          ; Plasma concentration residual error
    IF (CMT.EQ.3)    W = SQRT(SIGMA(2,2))          ; CSF concentration residual error
    IRES = IPRED – DV                              ; Individual residual error
    IWRES = IRES / W                               ; Individual weighted residual error
    IF (CMT.EQ.2)   Y = IPRED + EPS(1)             ; Plasma concentration additive residual error
    IF (CMT.EQ.3)   Y = IPRED + EPS(2)             ; CSF concentration additive residual error
```

```
        CMAX_P  = COM(1)                        ; Output plasma Cmax
        TMAX_P  = COM(2)                        ; Output plasma Tmax
        CMAX_C  = COM(3)                        ; Output CSF Cmax
        TMAX_C  = COM(4)                        ; Output CSF Tmax

$THETA                                          ; Initial estimates of theta
        (0, 40.7)                               ; 1. Clearance for subpopulation 1
        (0, 18.1)                               ; 2. Clearance for subpopulation 2
        (0, 96.7)                               ; 3. Central volume of distribution
        (0, 0.357)                              ; 4. Mean transit time
        (1) FIX                                 ; 5. Relative bioavailability
        (0, 0.0344)                             ; 6. Inter-compartment clearance
        (1) FIX                                 ; 7. Partition coefficient
        (0.15) FIX                              ; 8. CSF volume of distribution
        (0, 0.399)                              ; 9. Probability of subpopulation 2

$OMEGA                                          ; Initial estimates for omega
        0.0215                                  ; 1. Clearance
        0 FIX                                   ; 2. Central volume of distribution
        1.05                                    ; 3. Mean transit time
        0.212                                   ; 4. Relative bioavailability
        0.516                                   ; 5. Inter-compartment clearance
        0 FIX                                   ; 6. Partition coefficient
        0 FIX                                   ; 7. CSF volume of distribution

$SIGMA                                          ; Initial estimates of sigma
        (0.299)                                 ; 1. Plasma concentration residual variability
        (0.274)                                 ; 2. CSF concentration residual variability

$ESTIMATION   POSTHOC MAXEVAL=9999   METHOD=1 INTER
```

NONMEM code for final isoniazid population PD model

```
$INPUT
        ID              ; Patient ID
        TIME            ; Time of sample/dose
        DV              ; Dependent variable (1 = Death, 0 = survival)
        CMT             ; Compartment
        EVID            ; Event ID record
        HIV             ; Covariate (1 = HIV co-infection, 0 = no HIV co-infection)
        GCS             ; Covariate (Glasgow coma score)
        CMAXC           ; Isoniazid CSF maximum concentration (µg/L)

$DATA
        dataset.csv  IGNORE=#

$SUBROUTINE
        ADVAN6 TOL=3

$MODEL
        COMP = (1)              ; Hazard

$PK
;----------------------------------- Isoniazid CSF Cmax effect-------------------------------------------------------------------
        EMAX = THETA(5)                         ; Maximum effect on hazard
        EC50 = THETA(6)                         ; CSF Cmax to reach 50% of maximum effect
        HILL = THETA(7)                         ; Shape parameter
        EFF = 1 - EMAX*CMAXC**HILL/(CMAXC**HILL+EC50**HILL)
                                                ; CSF Cmax effect
;------------------------------------------------------------------------------------------------------------------------- -------------

;----------------------------------- GCS covariate---------------------------------------------------------------------------------
        CGCS =   1 + THETA(3)*(GCS-14)          ; Linear covariate relationship for GCS
;------------------------------------------------------------------------------------------------------------------------- -------------
```

```
;----------------------------------- HIV covariate-----------------------------------------------------------------------------------
    CHIV =   1 + THETA(4)*HIV                        ; Linear covariate relationship for HIV
;----------------------------------------------------------------------------------------------------------------------------------

    TVSIGCE  = THETA(1)                              ; Standard deviation of log-normal distribution
    SIGCE = TVSIGCE + ETA(1)                         ; Individual standard deviation
    TVMUCE   = THETA(2)  * CGCS * CHIV               ; Median of log-normal distribution
    MUCE = TVMUCE + ETA(2)                           ; Individual median
    PI = 3.1415                                      ; Pi value

$DES
    DEL   = 1E-16                                    ; Small number to avoid LOG(0)
    TIMX   = T+DEL                                   ; Time
    LTIMX  = LOG(TIMX)                               ; Natural Log transformation of time
    X2X    = (LTIMX-MUCE)/SIGCE                      ; Part 1 of log-normal distribution
    PDF2X  = EXP(-(1/2)*(X2X**2))/SQRT(2*PI)         ; Part 2 of log-normal distribution
    LOGCEX = ((1/(TIMX*SIGCE))*PDF2X/(1-PHI(X2X))) *EFF             ; Log-normal distribution
    DADT(1)= LOGCEX                                  ; Log-normal distribution hazard

$ERROR
    DE    = 1E-16                                    ; Small number to avoid LOG(0)
    TIM    = TIME+DE                                 ; Time
    LTIM   = LOG(TIM)                                ; Natural Log transformation of time
    CHZ   = A(1)                                     ; Cumulative hazard
    SUR   = EXP(-CHZ)                                ; Survival function

    X2     = (LTIM-MUCE)/SIGCE                       ; Part 1 of log-normal distribution
    PDF2   = EXP(-(1/2)*(X2**2))/SQRT(2*PI)          ; Part 2 of log-normal distribution
    LOGCE  = ((1/(TIM*SIGCE))*PDF2/(1-PHI(X2)))*EFF  ; Log-normal distribution
    HAZ    = LOGCE                                   ; Log-normal distribution hazard
```

```
        IF(DV.EQ.0) THEN
          PR = SUR                          ; Censored event (probability of survival)
         ENDIF
        IF (DV.EQ.1) THEN
          PR = SUR * HAZ                    ; Observed event
        ENDIF
        Y = PR
$THETA                                      ; Initial estimates of theta
        (0, 1.18)                           ; 1. Standard deviation
        (0, 3.42)                           ; 2. Median
        (0, 0.0907)                         ; 3. GCS effect on median
        (-0.5, -0.276)                      ; 4. HIV effect on median
        (0.99) FIX                          ; 5. Maximum effect of CSF Cmax
        (0, 1.37)                           ; 6. CSF Cmax to reach 50% of maximum effect
        (0, 2.8)                            ; 7. Shape parameter

$OMEGA                                      ; Initial estimates for omega
        0 FIX                               ; 1. Standard deviation
        0 FIX                               ; 2. Median

$ ESTIMATION MAXEVAL=9999 POSTHOC METHOD=1 MCETA=500 LAPLACIAN LIKE NUMERICAL
```
